# IP3R2-mediated Ca^2+^ release promotes LPS-induced cardiomyocyte pyroptosis via the activation of NLRP3/Caspase-1/GSDMD pathway

**DOI:** 10.1038/s41420-024-01840-8

**Published:** 2024-02-20

**Authors:** Qing-Rui Wu, Hui Yang, Hui-Dan Zhang, Yong-Jiang Cai, Yan-Xiang Zheng, Heng Fang, Zi-Fan Wang, Su-Juan Kuang, Fang Rao, Huan-Lei Huang, Chun-Yu Deng, Chun-Bo Chen

**Affiliations:** 1 School of Medicine, South China University of Technology, 510006 Guangzhou, China; 2 Guangdong Provincial Key Laboratory of Clinical Pharmacology, Research Center of Medical Sciences, Guangdong Provincial People’s Hospital, Guangdong Academy of Medical Sciences, Southern Medical University, 510080 Guangzhou, Guangdong China; 3 Department of Emergency Medicine, Qilu Hospital of Shandong University, 250012 Jinan, China; 4 School of Pharmaceutical Sciences, Southern Medical University, 510515 Guangzhou, China; 5 Department of Critical Care Medicine, Guangdong Provincial People’s Hospital (Guangdong Academy of Medical Sciences), Southern Medical University, Guangzhou, China; 6 Department of Cardiovascular Surgery, Guangdong Provincial People’s Hospital, Guangzhou, China; 7 Department of Critical Care Medicine, Shenzhen People’s Hospital, The Second Clinical Medical College of Jinan University, The First Affiliated Hospital of Southern University of Science and Technology, 518000 Shenzhen, Guangdong Province China

**Keywords:** Mechanisms of disease, Acute inflammation

## Abstract

Pyroptosis plays a crucial role in sepsis, and the abnormal handling of myocyte calcium (Ca^2+^) has been associated with cardiomyocyte pyroptosis. Specifically, the inositol 1,4,5-trisphosphate receptor type 2 (IP3R2) is a Ca^2+^ release channel in the endoplasmic reticulum (ER). However, the specific role of IP3R2 in sepsis-induced cardiomyopathy (SIC) has not yet been determined. Thus, this study aimed to investigate the underlying mechanism by which IP3R2 channel-mediated Ca^2+^ signaling contributes to lipopolysaccharide (LPS)—induced cardiac pyroptosis. The SIC model was established in rats by intraperitoneal injection of LPS (10 mg/kg). Cardiac dysfunction was assessed using echocardiography, and the protein expression of relevant signaling pathways was analyzed using ELISA, RT-qPCR, and western blot. Small interfering RNAs (siRNA) and an inhibitor were used to explore the role of IP3R2 in neonatal rat cardiomyocytes (NRCMs) stimulated by LPS in vitro. LPS-induced NLRP3 overexpression and GSDMD-mediated pyroptosis in the rats’ heart. Treatment with the NLRP3 inhibitor MCC950 alleviated LPS-induced cardiomyocyte pyroptosis. Furthermore, LPS increased ATP-induced intracellular Ca^2+^ release and IP3R2 expression in NRCMs. Inhibiting IP3R activity with xestospongin C (XeC) or knocking down IP3R2 reversed LPS-induced intracellular Ca^2+^ release. Additionally, inhibiting IP3R2 reversed LPS-induced pyroptosis by suppressing the NLRP3/Caspase-1/GSDMD pathway. We also found that ER stress and IP3R2-mediated Ca^2+^ release mutually regulated each other, contributing to cardiomyocyte pyroptosis. IP3R2 promotes NLRP3-mediated pyroptosis by regulating ER Ca^2+^ release, and the mutual regulation of IP3R2 and ER stress further promotes LPS-induced pyroptosis in cardiomyocytes.

## Introduction

Sepsis is a life-threatening condition characterized by organ dysfunction caused by an imbalanced immune response to infection [1, 2]. Sepsis-induced cardiomyopathy (SIC) is a common cardiac dysfunction observed in most sepsis patients, and it has long been an area of significant research interest [3]. Pyroptosis, triggered by inflammation and exacerbates inflammatory responses, is believed to play a crucial role in SIC [4]. The NOD-like receptor protein 3 (NLRP3) inflammasome/Caspase-1/ GSDMD pathway has been identified as a classical pathway involved in regulating pyroptosis [5], and recent studies have demonstrated that inhibition of GSDMD-dependent pyroptosis can improve cardiac dysfunction in septic cardiomyopathy [6]. In addition, the suppression of inflammation, cell apoptosis and pyroptosis by blocking Toll-like receptor 4 (TLR4) signaling and NLRP3 inflammasome pathways has been shown to alleviate myocardial dysfunction in sepsis [7]. However, the specific regulatory mechanisms that govern pyroptosis in cardiomyocytes remain unclear, and additional research is necessary to fully understand them.

The role of abnormal cellular calcium (Ca^2+^) handling in mediating NLRP3-mediated pyroptosis in SIC is not yet fully understood, despite the well-established significance of Ca^2+^ in various cardiomyocyte functions, such as muscle contraction, gene expression, and cell death. Previous studies have indicated that abnormal Ca^2+^ handling can contribute to NLRP3 inflammasome activation. For instance, Ca^2+^ flux-triggered NLRP3 inflammasome activation has been linked to postoperative atrial fibrillation [8], and inflammation and NLRP3 inflammasome activation driven by Ca^2+^ signaling have been implicated in heart failure [9]. Thus, inhibiting the Ca^2+^-dependent activation of NLRP3 inflammasomes and reducing cardiac inflammation have been shown to be beneficial in treating heart failure [10]. However, the specific role and underlying mechanism by which abnormal cellular Ca^2+^ handling contributes to NLRP3-mediated pyroptosis in SIC remain unknown.

Inositol (1,4,5) trisphosphate receptors (IP3Rs) are Ca^2+^ release channels localized within the endoplasmic reticulum (ER), which is the major Ca^2+^ store in the cells. There are three isoforms of IP3Rs: IP3R1, IP3R2 and IP3R3, encoded by different genes. Among them, IP3R2 is the predominant isoform expressed in cardiac cells [11]. IP3R activity is crucial for maintaining Ca^2+^ homeostasis and cell survival. Studies have shown that inhibiting IP3R2-mediated Ca^2+^ oscillations could be a novel anti-tumor strategy against liver cancer [12]. Although the role of IP3R2 in SIC and its underlying mechanism remain unknown, previous research has demonstrated that inhibiting ER stress can alleviate septic cardiomyopathy [13, 14]. Additionally, ER stress has been shown to activate the NLRP3 inflammasome, leading to pyroptotic cell death in atherosclerosis [15], and the inhibition of ER stress has also been found to improve cardiac function in isoproterenol-induced heart failure rats by suppressing cardiomyocyte pyroptosis [16]. However, there is no evidence regarding the impact of ER stress on IP3R2 regarding septic cardiomyopathy. Based on this, we hypothesized that ER stress might contribute to cardiomyocyte pyroptosis by activating IP3R2-mediated Ca^2+^ release.

In this study, we utilized lipopolysaccharide (LPS) to construct a sepsis rat model and LPS treatment of neonatal rat cardiomyocytes (NRCMs) to explore the mechanism of IP3R2 regulating intracellular Ca^2+^ dynamics and Ca^2+^ mediated pyroptosis in cardiomyocytes. We demonstrate that IP3R2 promotes NLRP3-mediated pyroptosis by regulating ER Ca^2+^ release, and the mutual regulation of IP3R2 and ER stress further promotes LPS-induced pyroptosis in cardiomyocytes. Herein, suppressing IP3R2/NLRP3 signaling pathway may be a promising therapeutic option to effectively depress SIC.

## Results

### LPS-induced NLRP3 overexpression and GSDMD-mediated pyroptosis in the heart

**Fig. 1:**
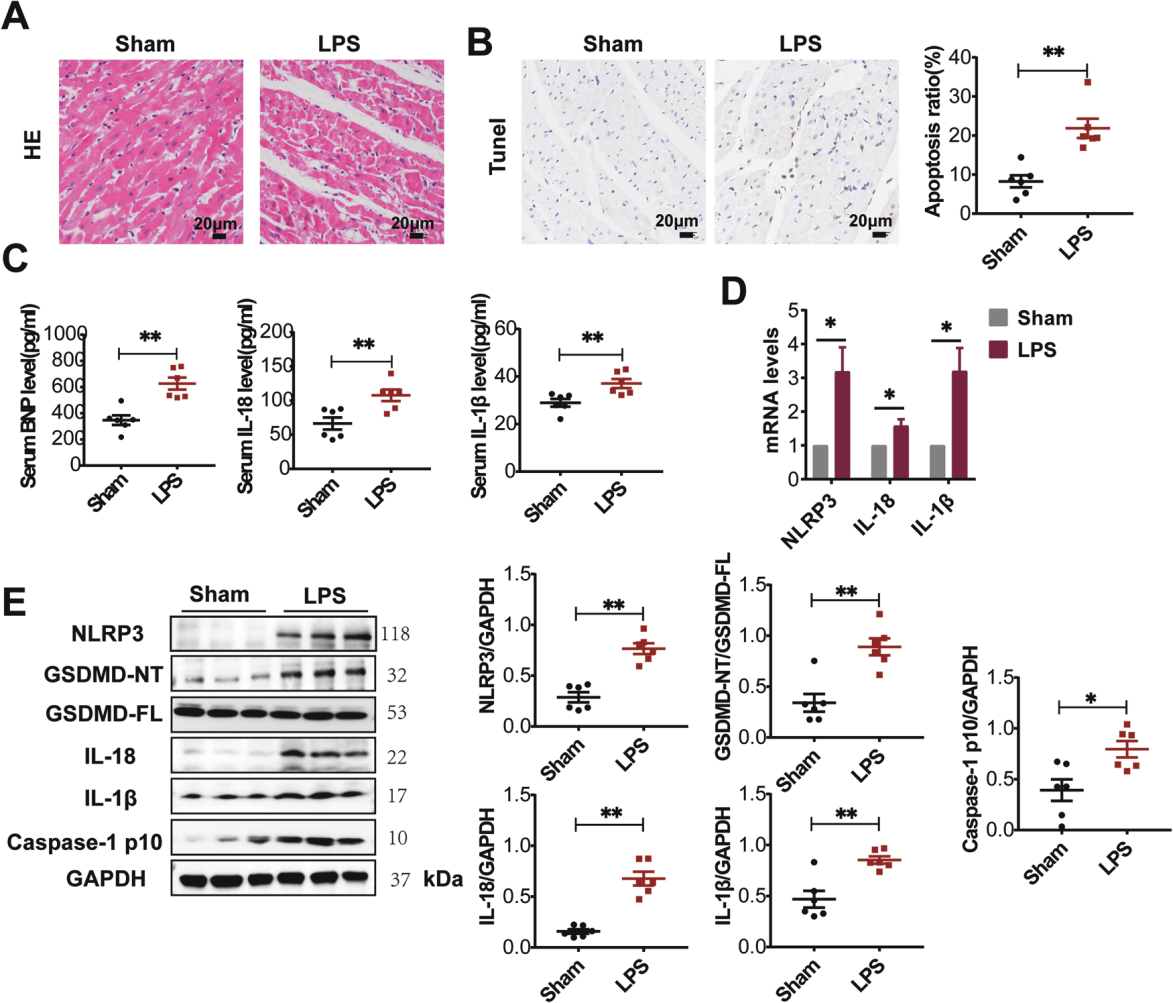
LPS induces the activation of NLRP3 inflammasome and cardiac pyroptosis in rats. **A** The representative images of the left ventricle tissues of rats stained with HE (*n* = 6). **B** Apoptosis in myocardial tissues was detected by TUNEL staining (*n* = 6). **C** ELISA was used to detect serum BNP, IL-18 and IL-1β levels in the rats of each group (*n* = 6). **D** NLRP3, IL-18 and IL-1β mRNA levels were detected in the left ventricle tissues of rats using RT-qPCR analysis (*n* = 4). **E** Western blotting images and the summarized data showing NLRP3, GSDMD-FL, GSDMD-NT, Caspase-1 p10, IL-18 and IL-1β levels in the ventricular tissues of sham and LPS rats (*n* = 6). Data are shown as mean ± SEM. ^*^P < 0.05, ^**^P < 0.01.

LPS-induced septic cardiomyopathy models were established by intraperitoneally injecting LPS (10 mg/kg, 6 h) into rats. Echocardiographic analysis revealed that the LPS group exhibited cardiac dysfunction characterized by decreased LVEF, LVFS and SV (Table 1). Histological examination of myocardial tissue using hematoxylin-eosin (HE) staining showed disordered cardiac muscle fibers and increased cell gap in the LPS group compared to the sham group (Fig. 1A). TUNEL staining of myocardial tissue indicated increased apoptosis in the LPS group (Fig. 1B). ELISA analysis demonstrated significantly elevated levels of serum brain natriuretic peptide (BNP) and inflammatory factors (IL-1β and IL-18) in the LPS group compared to the sham group (Fig. 1C). Moreover, RT-qPCR analysis revealed higher mRNA levels of NLRP3, IL-1β, and IL-18 in the myocardial tissue of the LPS group compared to the sham group (Fig. 1D). Notably, protein expression levels of NLRP3, Caspase-1 p10, IL-1β, IL-18, and the N-terminus of GSDMD (GSDMD-NT) were increased in the myocardial tissue of the LPS group rats compared to the sham group (Fig. 1E). Taken together, these findings suggest that LPS-induced cardiac dysfunction could be associated with the upregulation of NLRP3, leading to GSDMD-mediated pyroptosis in the myocardial tissue.

**Table 1:** LPS-induced cardiac dysfunction in rats.

Group	Sham (*n* = 6)	LPS (*n* = 6)
LVIDs (mm)	4.3 ± 0.5	4.6 ± 0.4
LVIDd (mm)	6.3 ± 0.3	6.1 ± 0.4
LVs (μl)	82.6 ± 20.3	98.5 ± 22.5
LVd (μl)	200.4 ± 23.1	190.9 ± 25.0
SV (μl)	117.9 ± 8.7	92.4 ± 12.3^**^
LVEF (%)	59.3 ± 6.1	48.8 ± 6.1^*^
LVFS (%)	32.4 ± 4.2	25.3 ± 3.8^*^
CO (ml/min)	40.4 ± 4.3	34.9 ± 5.1
LVAWs (mm)	2.3 ± 0.1	2.3 ± 0.2
LVAWd (mm)	1.6 ± 0.1	1.7 ± 0.1
LVPWs (mm)	2.3 ± 0.2	2.3 ± 0.1
LVPWd (mm)	1.7 ± 0.2	1.9 ± 0.1

All data are shown as mean ± SEM. *p < 0.05, **p < 0.01, vs. Sham group.

*LVIDs* left ventricular end-systolic internal dimension, *LVIDd* left ventricular end-diastolic internal dimension, *LVs* left ventricular systolic volume, *LVd* left ventricular diastolic volume, *SV* stroke volume, *LVEF (%)* left ventricular ejection fraction, *LVFS (%)* left ventricular fraction shortening, *CO* cardiac output, *LVAWs* left ventricular end-systolic anterior wall thickness, *LVAWd* left ventricular end-diastolic anterior wall thickness, *LVPWs* left ventricular end-systolic posterior wall thickness, *LVPWd* left ventricular end-diastolic posterior wall thickness.

### MCC950 prevents NLRP3-mediated pyroptosis in NRCMs-induced by LPS

**Fig. 2:**
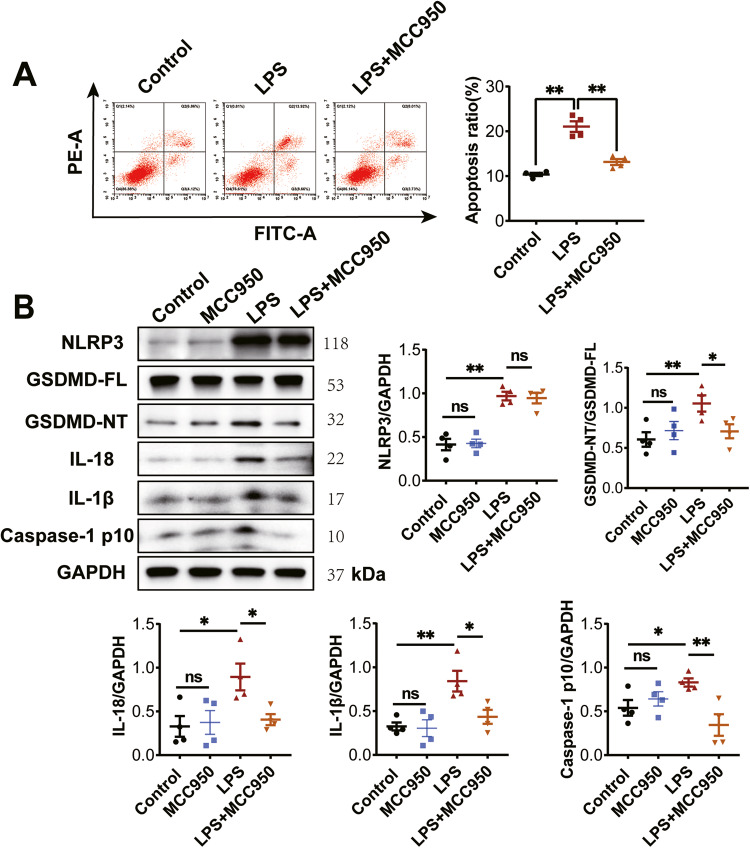
LPS induces cardiomyocyte pyroptosis by activating NLRP3 inflammasome and Caspase-1. **A** NRCMs were stained with Annexin V/PI and subjected to flow cytometry to detect apoptosis in NRCMs (*n* = 4). **B** Western blotting images and the summarized data showing the effect of MCC950 (10 μM) on the protein expression of NLRP3, GSDMD-NT, Caspase-1 p10, IL-18 and IL-1β (*n* = 4) in NRCMs-induced by LPS. Data are shown as mean ± SEM. ^*^P < 0.05, ^**^P < 0.01. ns: no significant statistical difference.

To investigate the role of NLRP3 in inducing GSDMD-mediated pyroptosis, NRCMs were pre-treated with an NLRP3 inhibitor, MCC950 (10 μM), followed by stimulation with LPS (2 μg/ml) for 24 h. Consistent with the in vivo findings, flow cytometry analysis demonstrated that LPS promoted apoptosis in NRCMs, while pre-treatment with MCC950 significantly reversed the apoptosis ratio (Fig. 2A). Western blot analysis revealed that MCC950 pre-treatment did not affect the induction of NLRP3 by LPS in NRCMs. However, MCC950 pre-treatment significantly inhibited the expression of GSDMD-NT, Caspase-1 p10, IL-1β, and IL-18 induced by LPS in NRCMs (Fig. 2B). These results suggest that NLRP3 is involved in GSDMD-mediated pyroptosis by activating Caspase-1 in LPS-induced NRCMs.

### LPS increases ATP-induced intracellular Ca^2+^ Release and the expression of IP3R2 in NRCMs

**Fig. 3:**
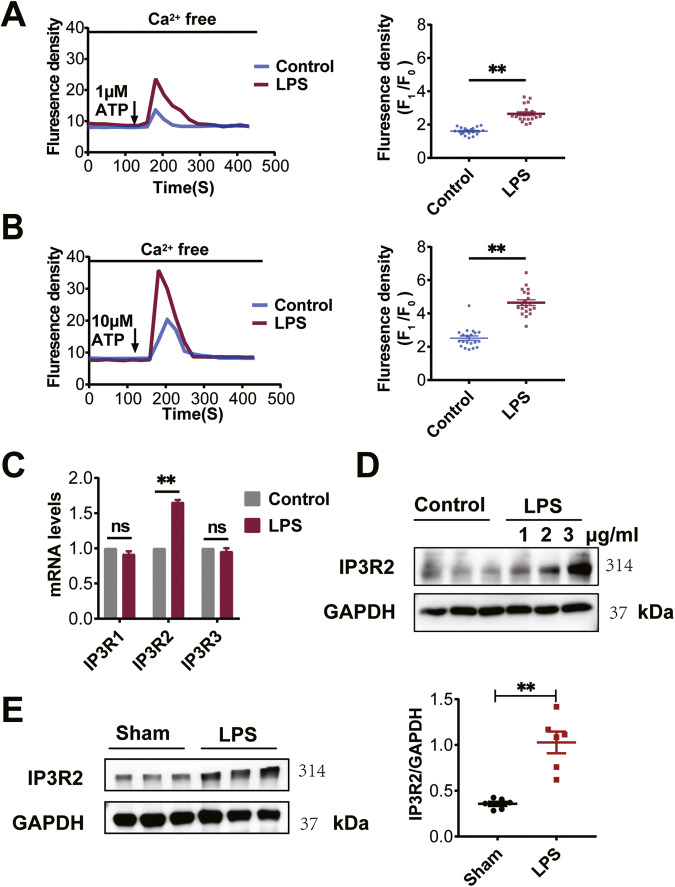
LPS increases ATP-induced intracellular Ca^2+^ release and the expression of IP3R2 in NRCMs. **A**, **B** Representative traces of Fluo4/AM changes over time by adding of 1 μM ATP or 10 μM ATP, respectively (left). Scatter plot showing the ATP-evoked calcium peak amplitude after adding 1 μM ATP or 10 μM ATP (right) (*n* = 20 cells). **C** IP3R1, IP3R2 and IP3R3 mRNA levels in NRCMs detected via RT-qPCR analysis (*n* = 4). **D** Western blotting images showing the expression of IP3R2 protein in NRCMs increased with an increase in LPS concentration. **E** Western blotting images and summarized data showing the IP3R2 protein expression level significantly increased in the myocardium of LPS group rats (*n* = 6). Data are shown as mean ± SEM. ^**^P < 0.01. ns: no significant statistical difference.

To investigate the mechanism by which LPS treatment promotes the activation of NLRP3 inflammasomes, we examined the intracellular Ca^2+^ release using ATP, a well-known activator of IP3R-mediated Ca^2+^ release [17]. Fluo-4 assay results demonstrated that NRCMs, after incubation in a calcium-free buffer for 2 min, exhibited increased Ca^2+^ transients upon stimulation with 1 μM ATP (Fig. 3A) or 10 μM ATP (Fig. 3B) in the LPS group compared to the control group. The rise in Ca^2+^ transients was more pronounced at 10 μM ATP. To investigate which isoform of IP3R is involved in the LPS-induced Ca^2+^ release, we examined the mRNA expression levels of the three IP3R subtypes (IP3R1, IP3R2, and IP3R3) in both control and LPS-treated groups. The results revealed no significant difference in the mRNA levels of IP3R1 and IP3R3 between the control and LPS groups in NRCMs, while the mRNA levels of IP3R2 were upregulated in the LPS group (Fig. 3C). Furthermore, western blot analysis showed increased expression of IP3R2 protein in NRCMs with increasing LPS concentration (Fig. 3D). Consistently, the protein expression level of IP3R2 was significantly elevated in the myocardial tissue of rats in the LPS group (Fig. 3E). These findings suggest that LPS-induced intracellular Ca^2+^ release may be associated with the upregulation of IP3R2.

### IP3R2-mediated Ca^2+^ release is involved in LPS-induced pyroptosis of cardiomyocyte

**Fig. 4:**
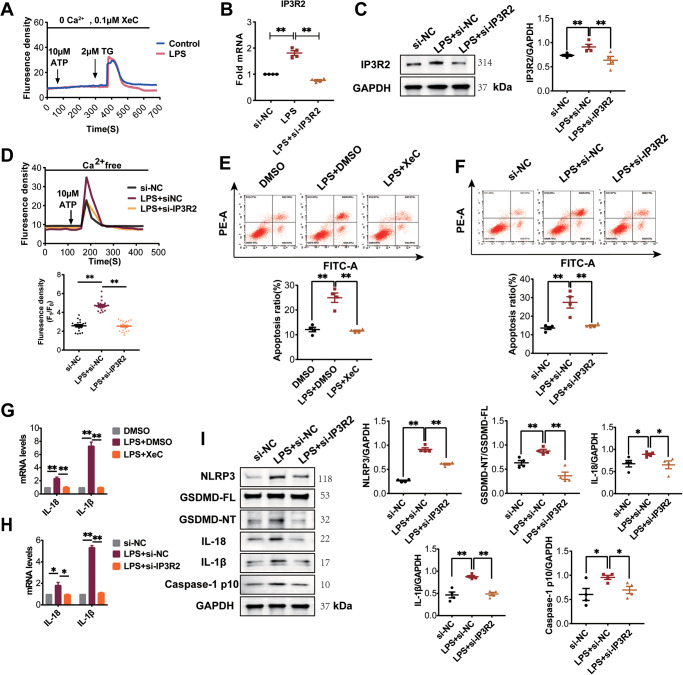
IP3R2-mediated Ca^2+^ release is involved in LPS-induced pyroptosis of cardiomyocytes. **A** NRCMs were pre-treated with IP3R inhibitor XeC (0.1 μM) and monitored for changes in intracellular Ca^2+^ levels stimulated by ATP (10 μM). **B** Detection of IP3R2 mRNA levels in NRCMs with si-IP3R2 intervention using RT-qPCR analysis (*n* = 4). **C** Western blotting images and the summarized data showing the IP3R2 protein expression level significantly decreased in NRCMs with si-IP3R2 intervention (*n* = 4). **D** Representative traces of Fluo4/AM changes over time by adding of 10 μM ATP (left). Scatter plot showing the ATP-evoked calcium peak amplitude after adding ATP (right) (*n* = 20 cells). **E**, **F** Flow cytometry analysis was conducted to examine the effects of XeC or si-IP3R2 on apoptosis in NRCMs induced by LPS (*n* = 4). **G**, **H** IL-18 and IL-1β mRNA levels were detected in NRCMs with XeC or si-IP3R2 intervention using RT-qPCR analysis (*n* = 4). **I** Western blotting images and summarized data showing the protein expression level of NLRP3, GSDMD-NT, Caspase-1 p10, IL-18 and IL-1β (*n* = 4) significantly decreased in NRCMs with si-IP3R2 intervention. Data are shown as mean ± SEM. ^*^P < 0.05, ^**^P < 0.01.

To further confirm the involvement of IP3R2 in LPS-induced intracellular Ca^2+^ release, NRCMs were pre-treated with the IP3R inhibitor xestospongin C (XeC) and monitored for changes in intracellular Ca^2+^ levels stimulated by ATP. We observed that XeC completely inhibited the ATP-induced Ca^2+^ increase in both the control and LPS groups, while thapsigargin (TG) triggered Ca^2+^ release from the ER, indicating that the increase in Ca^2+^ release from the ER is mediated by IP3R (Fig. 4A). Furthermore, to evaluate the specific role of IP3R2 channels in LPS-induced intracellular Ca^2+^ release, we used small interfering RNA (siRNA) to knock down IP3R2. The results showed that the mRNA and protein expression levels of IP3R2 were significantly increased in NRCMs stimulated with LPS, and this increase was effectively blocked when NRCMs were treated with LPS and si-IP3R2 (Fig. 4B, C). Additionally, IP3R2 knockdown reversed the LPS-induced intracellular Ca^2+^ release, suggesting that IP3R2, rather than IP3R1 or IP3R3, mediates the ER Ca^2+^ release induced by LPS (Fig. 4D). Flow cytometry analysis revealed that both XeC and si-IP3R2 significantly attenuated the LPS-induced increase in cell apoptosis (Fig. 4E, F). Moreover, both XeC and si-IP3R2 inhibited the mRNA levels of IL-1β and IL-18 in LPS-treated NRCMs (Fig. 4G, H). Western blot analysis demonstrated that downregulation of IP3R2 significantly inhibited the LPS-induced increase in the expression of NLRP3, GSDMD-NT, Caspase-1 p10, IL-1β, and IL-18 in NRCMs (Fig. 4I). Collectively, these findings suggest that IP3R2-mediated intracellular Ca^2+^ release regulates LPS-induced cardiomyocyte pyroptosis through the NLRP3/Caspase-1/GSDMD pathway.

### Inhibition of ER stress improves LPS‐induced pyroptosis of cardiomyocyte via NLPR3/ Caspase-1 pathway

**Fig. 5:**
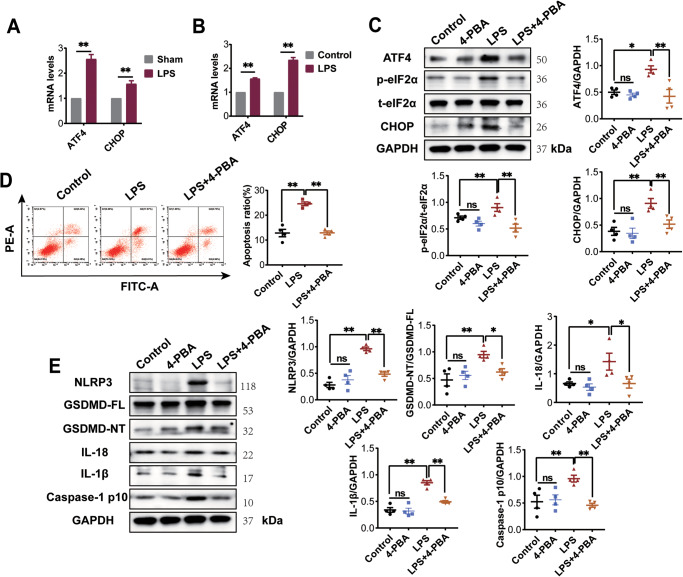
Inhibition of ER stress improves LPS‐induced pyroptosis of cardiomyocytes via the NLPR3/Caspase-1 pathway. **A**, **B** Detection of ATF4 and CHOP mRNA levels in rat myocardial tissues and NRCMs via RT-qPCR analysis (*n* = 4). **C** Western blotting images and the summarized data showing the protein expression level of p-eIF2α, CHOP and ATF4 (*n* = 4) significantly decreased in NRCMs with 4-PBA intervention (5 mM). **D** Flow cytometry analysis was conducted to examine the effects of 4-PBA on apoptosis in NRCMs induced by LPS. **E** Western blotting images and the summarized data showing the protein expression level of NLRP3, GSDMD-NT, Caspase-1 p10, IL-18 and IL-1β significantly decreased in NRCMs with 4-PBA intervention (*n* = 4). Data are shown as mean ± SEM. ^*^P < 0.05, ^**^P < 0.01. ns: no significant statistical difference.

We observed that LPS induced the mRNA expression of ER stress markers ATF4 and CHOP in both rat myocardial tissue and NRCMs (Fig. 5A, B). To investigate the role of ER stress in LPS-induced cardiomyocyte pyroptosis, NRCMs were treated with 4-phenylbutyrate (4-PBA), an ER stress antagonist [18]. According to the results in the pre-experiment, 5 mM 4-PBA treatment was chosen for subsequent experiments (Supplementary Fig. S2). Consistent with the increased mRNA levels, the protein levels of ER stress markers p-eIF2α, ATF4, and CHOP were elevated in the LPS group. However, administration of 4-PBA significantly reduced the protein levels of p-eIF2α, ATF4, and CHOP (Fig. 5C). These findings suggest that 4-PBA effectively inhibits LPS-induced ER stress in cardiomyocytes. Furthermore, flow cytometry analysis demonstrated that 4-PBA significantly attenuated the increased apoptosis in LPS-induced NRCMs (Fig. 5D). Western blot results revealed that 4-PBA antagonized the LPS-induced increase in protein levels of NLRP3, GSDMD-NT, Caspase-1 p10, IL-18, and IL-1β (Fig. 5E). Taken together, these results indicate that ER stress plays a role in LPS-induced cardiomyocyte pyroptosis through the NLRP3 inflammasome-mediated activation of Caspase-1.

### The mutual promotion relationships between IP3R2 and ER stress aggravates LPS induced pyroptosis of cardiomyocyte

**Fig. 6:**
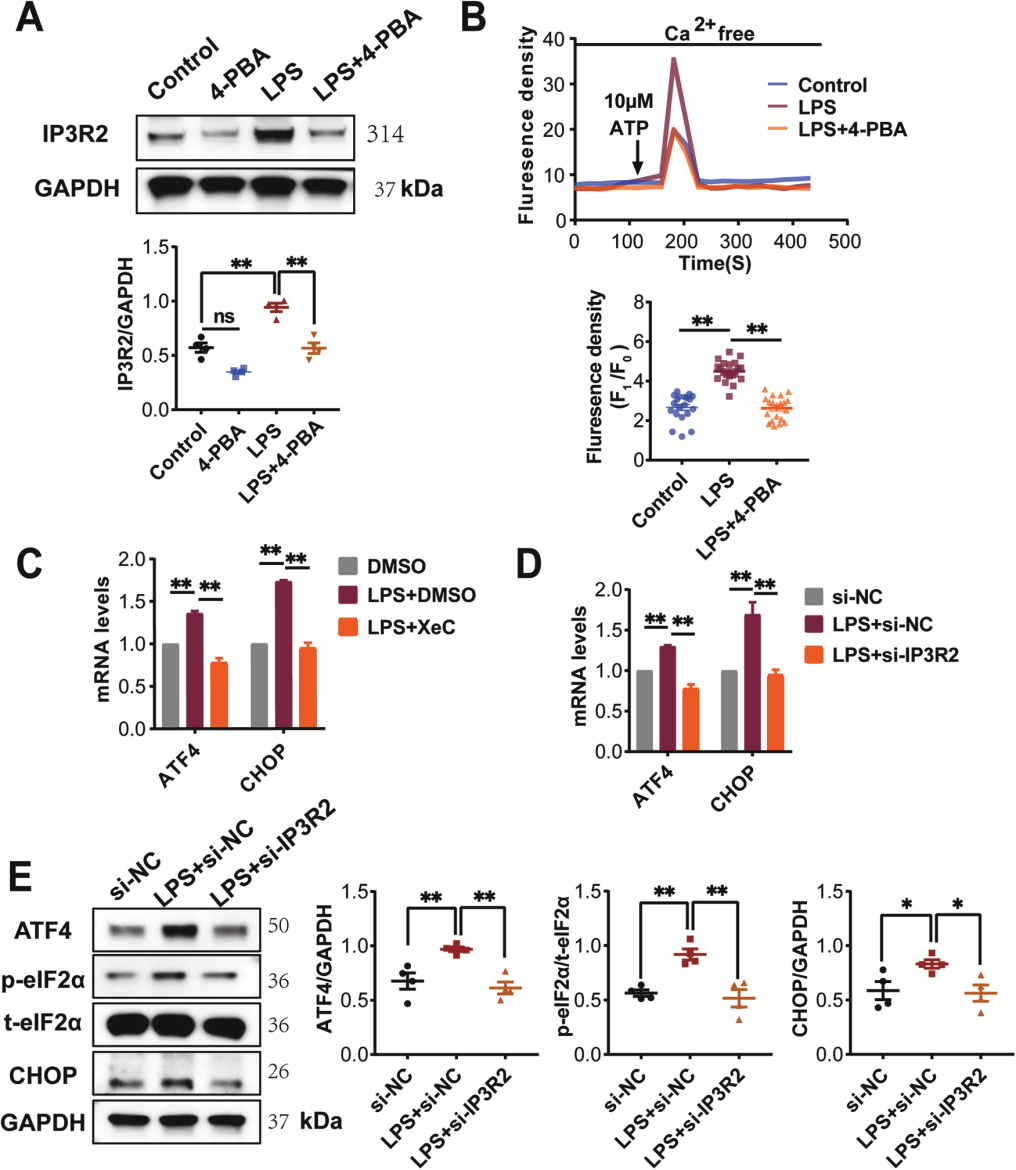
The mutual promotion relationships between IP3R2 and ER stress aggravates LPS induced pyroptosis of cardiomyocytes. **A** Western blotting images and the summarized data showing IP3R2 protein expression level significantly decreased in NRCMs following 4-PBA intervention (*n* = 4). **B** Representative traces of Fluo-4/AM changes over time after adding 10 μM ATP (left). Scatter plot showing the ATP-evoked calcium peak amplitude after adding ATP (right) (*n* = 20 cells). **C**, **D** Detection of ATF4 and CHOP mRNA levels in NRCMs with XeC or si-IP3R2 intervention via RT-qPCR analysis (*n* = 4). **E** Western blotting images and the summarized data showing the protein expression level of p-eIF2α, CHOP and ATF4 (*n* = 4) significantly decreased in NRCMs following si-IP3R2 intervention. Data are shown as mean ± SEM. ^*^P < 0.05, ^**^P < 0.01. ns: no significant statistical difference.

To further investigate the mechanism of LPS-induced IP3R2 activation, we examined the effect of 4-PBA on the expression and function of IP3R2 in LPS-induced NRCMs. Interestingly, 4-PBA inhibited the LPS-induced increase in IP3R2 expression and intracellular Ca^2+^ release (Fig. 6A, B). This suggests that inhibition of ER stress prevents LPS-induced cardiomyocyte pyroptosis, potentially by downregulating IP3R2-mediated intracellular Ca^2+^ release in NRCMs. Although we established that ER stress promotes IP3R2-mediated Ca^2+^ release in LPS induced pyroptosis, it is unclear whether IP3R2 regulates ER stress. To address this, we further investigated the effects of IP3R2 inhibition on ER stress. Intriguingly, both XeC and si-IP3R2, which inhibit IP3R2 function and expression, respectively, prevented the increase in mRNA levels of ATF4 and CHOP in LPS-induced NRCMs (Fig. 6C, D). Consistent with the mRNA level experiments, si-IP3R2-mediated inhibition of IP3R2 reduced the expression of LPS-induced p-eIF2α, ATF4, and CHOP proteins (Fig. 6E). These results suggest that IP3R2 is involved in LPS-promoted ER stress by mediating Ca^2+^ release. Overall, our findings indicate the existence of a mutually positive feedback loop between IP3R2 and ER stress that exacerbates cardiomyocyte pyroptosis and that IP3R2 could be a promising therapeutic target for LPS-induced myocardial injury.

## Discussion

**Fig. 7:**
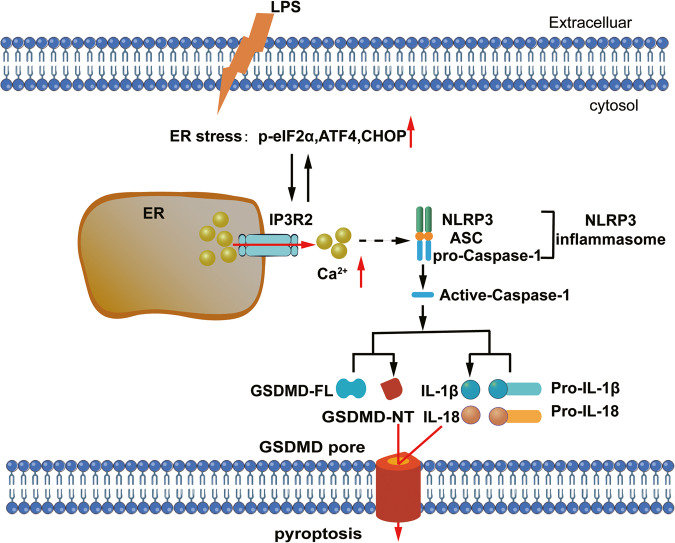
Schematic diagram depicting the proposed IP3R2-mediated Ca2+ signals in cardiomyocyte pyroptosis induced by LPS. LPS stimulation induces IP3R2 upregulation in NRCMs. Then, IP3R2-mediated Ca2+ release participates in LPS induced-cardiomyocytes pyroptosis. NLRP3 inflammasome is activated by IP3R-mediated Ca2+ release, characterized by upregulated NLRP3 expression. The activation of NLRP3 inflammasome governs the cleavage and activation of pro-Caspase-1. Activated Caspase-1 cleaves the full-length gasdermin D (GSDMD-FL) protein to release the N-terminal of GSDMD (GSDMD-NT), which results in GSDMD pore formation. Meanwhile, mature IL-18 and IL-1β are cleaved by the activated NLRP3/Caspase-1 pathway, and the cleaved IL-18 and IL-1β are released through the GSDMD pore in the cell membrane and induces pyroptosis. It is worth mentioning that the mutual regulation of IP3R2 and ER stress further exacerbates the pyroptosis process. Thus, inhibiting IP3R2 could be a promising strategy for the prevention of SIC.

The present study has highlighted the important role of IP3R2-mediated intracellular Ca^2+^ release in LPS-induced cardiomyocyte pyroptosis. Through pharmacological inhibition or siRNA-mediated knockdown of IP3R2, we observed decreased inflammatory response upon LPS stimulation. Additionally, inhibiting NLRP3 in NRCMs effectively prevented the activation of GSDMD induced by LPS. Notably, the inhibition of IP3R2 also inhibited the LPS-mediated activation of the NLRP3/Caspase-1/GSDMD pathway. Importantly, we found a close association between IP3R2-mediated Ca^2+^ release and ER stress, where both factors mutually induce and promote each other, thereby exacerbating LPS-induced cardiomyocyte pyroptosis. Consequently, the mutual activation of IP3R2 and ER stress contributes to the development of cardiac impairment by triggering the activation of the NLRP3/Caspase-1/GSDMD pathway (Fig. 7).

Previous studies have reported that mouse cardiac function decreases significantly after 6 and 12 h of intraperitoneal injection of LPS, then gradually recovers [19, 20]. Other research demonstrated that injecting 20 mg/kg LPS for 6 h can induce sepsis-induced cardiac dysfunction in mice [21]. In this study, we found that intraperitoneal injection of LPS (10 mg/kg) for 6 h resulted in significant cardiac dysfunction in rats, while treatment of NRCMs with LPS (2 μg/ml) for 24 h promoted cardiomyocyte apoptosis and inflammation. There has been increasing interest in the scientific community on GSDMD-mediated pyroptosis in LPS-cardiac dysfunction. Recent studies have revealed that the natural extract of syringaresinol attenuates sepsis-induced cardiac dysfunction by inhibiting inflammation and pyroptosis in mice [22]. Furthermore, inhibition of the NLRP3 inflammasome has been shown to alleviate cardiac dysfunction by inhibiting LPS-induced cardiomyocyte pyroptosis [23]. Consistent with these studies, our findings demonstrate that LPS promotes pyroptosis in an NLRP3-dependent manner, as pre-treatment with the NLRP3 inhibitor MCC950 can reverse LPS-induced GSDMD cleavage and cell death. As previously mentioned, MCC950 does not directly target NLRP3 inflammasome activation at the genetic level but rather directly bind to the NLRP3 NACHT domain, thereby preventing it from maintaining its active structural conformation. It inhibits cleavage of Caspase-1, IL-1β and IL-18 by inhibiting the oligomerization of associated speck-like protein (ASC) [24–28]. This is consistent with our experimental results. In our experiment, we found that MCC950 inhibits LPS-induced ASC oligomerization and prevents cleavage of Caspase-1, IL-1β and IL-18 in NRCMs (Supplementary Figure S1,3). Therefore, we demonstrated that MCC950 inhibits ASC oligomerization, thereby blocking NLRP3 inflammasome assembly and ultimately inhibiting inflammasome activity in NRCMs.

To further investigate the mechanism of NLRP3 activation, we examined changes in intracellular Ca^2+^ concentration. Our findings revealed that LPS treatment induced increased Ca^2+^ oscillations and intracellular Ca^2+^ release. Previous studies have primarily focused on the role of IP3R2 in mediating Ca^2+^ release from the ER, which can contribute to cell apoptosis, senescence, or death [29, 30]. However, the specific role of IP3R2 in regulating LPS-induced inflammatory injury in cardiomyocytes has not been previously investigated. Interestingly, we found that the expression of IP3R2 was significantly increased in the cardiac tissues of LPS-treated rats and LPS-treated NRCMs. Further investigation demonstrated that inhibition of IP3R2 significantly suppressed cell apoptosis and inflammation affected by LPS, suggesting a potential therapeutic target for LPS-induced cardiomyopathy.

A previous study revealed that IP3R2 is necessary for generating long-term and regular Ca^2+^ oscillations in cells, whereas IP3R1 is involved in irregular Ca^2+^ oscillations, and IP3R3 exhibits anti-oscillatory effects [31]. Another study showed that targeted inhibition of IP3R2 eliminates arrhythmogenic Ca^2+^ signaling in mouse atrial myocytes induced by endothelin-1 [32]. In our experiments, we observed a significant increase in the expression of IP3R2 following LPS stimulation, while the expression of IP3R1 and IP3R3 did not show significant changes. Moreover, siRNA-mediated IP3R2 inhibition abolished intracellular Ca^2+^ release induced by LPS. These findings further confirm the prominent role of IP3R2 in regulating Ca^2+^ oscillations in NRCMs under LPS stimulation.

As mentioned earlier, the inhibition of NLRP3 by MCC950 effectively suppressed GSDMD-mediated pyroptosis in LPS-treated NRCMs. Previous studies have indicated that increased intracellular Ca^2+^ is crucial for activating the NLRP3 inflammasome [33]. However, the precise mechanism by which Ca^2+^ is involved in the NLRP3 inflammasome pathway remains largely unclear. In this study, we hypothesized that IP3R2-mediated intracellular Ca^2+^ release promotes NLRP3 activation in LPS-treated cardiomyocytes. Our findings support this hypothesis, as we demonstrated that inhibition of the IP3R2 channel by XeC or si-IP3R2 abolished the LPS-induced increase in intracellular Ca^2+^ concentration and NLRP3 expression. Furthermore, we observed that si-IP3R2 significantly inhibited LPS-induced GSDMD cleavage, Caspase-1 activation, and the expression of inflammatory cytokines. These results provide strong evidence that IP3R2-mediated intracellular Ca^2+^ release promotes LPS-induced cardiomyocyte pyroptosis through the NLRP3/Caspase-1/GSDMD signaling pathway.

Previous studies have shown that LPS can induce ER stress and Ca^2+^ release while inhibiting ER stress-mediated NLRP3 inflammasome activation, alleviating acute lung injury [34]. Additionally, ER stress has been found to trigger the release of mitochondrial ROS and the production of NLRP3, thereby inhibiting NLRP3 inflammasome-mediated pyroptosis and improving atherosclerotic heart disease [15]. Consistent with previous findings, our study demonstrated that the ER stress inhibitor 4-PBA could effectively inhibit LPS-induced cardiomyocyte pyroptosis by targeting the NLRP3/Caspase-1/GSDMD pathway. Other studies have shown that ER stress-induced necroptosis in cardiac IR injury via the Ca^2+^ overload pathways [35]. However, it has also been shown that IP3R-controlled Ca^2+^ release could induce hepatic ER stress [36]. Also, ER Ca^2+^ release potentiates the ER stress and cell death in MCF-7 cells caused by oxidative stress [37]. However, the relationship between IP3R2-mediated intracellular Ca^2+^ release and ER stress in LPS-induced cardiomyopathy is unclear. Our experiments revealed that 4-PBA significantly reduced the LPS-induced up-regulation of IP3R2 and intracellular Ca^2+^ release in NRCMs. Conversely, the knockdown of IP3R2 expression inhibits the ER stress induced by LPS. These findings suggest a mutually positive feedback loop between IP3R2 and ER stress, exacerbating LPS-induced cardiomyocyte pyroptosis.

In conclusion, our study demonstrates that IP3R2 overexpression is a key characteristic of LPS-induced cardiac dysfunction. Further, using si-RNA transfection technology and chemical inhibitor, we showed that IP3R2-mediated Ca^2+^ release contributes to cardiomyocyte pyroptosis induced by LPS through the NLRP3/Caspase-1/GSDMD pathway, which could be further enhanced by the mutual regulation of IP3R2 and ER stress. Taken together, these results suggest that inhibiting IP3R2 could be a promising strategy for the prevention of SIC.

## Materials and methods

### Animals and treatment

Twelve male Sprague–Dawley (SD) rats (220–250 g) were obtained from the Experimental Animal Center of Nanfang Hospital, Southern Medical University of China, and randomly divided into two groups: the Sham group (*n* = 6) and the LPS group (*n* = 6). Prior to the experiments, the rats were housed under controlled conditions with a 12-h light/12-h dark cycle. The rats in the LPS group received an intraperitoneal injection (I.P) of 10 mg/kg LPS, while the Sham group received an equivalent volume of sterile saline. For the I.P. administration, LPS (Sigma) was dissolved in 0.9% normal saline to achieve a final dosage of 10 mg/kg. Six hours after the injection, Doppler echocardiography was performed to assess cardiac function in both groups. Subsequently, the rats were sacrificed, and left ventricular tissue and serum samples were collected for further analysis. The animal experimental procedures were conducted in accordance with the guidelines and regulations of the Laboratory Animal Ethics Committee of Guangdong Provincial People’s Hospital (ethics protocol number: KY-Z-2021-581-01).

### Echocardiography

Doppler echocardiography was used to evaluate cardiac function of rats, as described in our previous study [38]. Briefly, following anesthetization of the rats with 3% isoflurane, chest echocardiography was performed using the Vevo 2100 system (VisualSonics, Toronto, Canada) with a 21-MHz transducer. The cardiac function parameters are measured based on M-type tracing images or calculated through built-in software packages. The recorded parameters were as follows: left ventricular end-systolic internal dimension (LVIDs), left ventricular end-diastolic internal dimension (LVIDd), left ventricular systolic volume (LVs), left ventricular diastolic volume (LVd), Stroke Volume(SV), left ventricular ejection fraction (%LVEF), left ventricular fraction shortening (%LVFS), Cardiac Output (CO), left ventricular end-systolic anterior wall thickness (LVAWs), left ventricular end-diastolic anterior wall thickness (LVAWd), left ventricular end-systolic posterior wall thickness (LVPWs), left ventricular end-diastolic posterior wall thickness (LVPWd).

### Cell culture and treatment

Primary neonatal rat cardiomyocytes (NRCMs) were isolated and purified from neonatal Sprague-Dawley (SD) rats using the method described in our previous study [38]. The NRCMs were cultured in DEMEM/F12 medium (Gibco) supplemented with 10% fetal bovine serum and 1% penicillin/streptomycin (Gibco). The cells were maintained in a humidified incubator at 37 °C with 95% O_2_ and 5% CO_2_. Before the experiments, the NRCMs were serum-starved for 3 h, and then the cells were pre-treated with specific inhibitors (10 μM MCC950, 0.1 μM XeC, and 5 mM 4-PBA) for 1 h before being stimulated with LPS (2 μg/ml) for 24 h. The inhibitors MCC950, XeC, and 4-PBA were procured from MedChemExpress (MCE).

### ELISA analysis

We isolated the rat serum from whole blood, and the levels of cytokines (IL-1β and IL-18) and brain natriuretic peptide (BNP) were measured using ELISA kits following the manufacturer’s instructions. The ELISA kits used in this study were purchased from Huamei Biological Engineering Co. Ltd. (Wuhan, China).

### Histological analysis

The rat myocardial tissues were fixed by perfusing them with a 4% paraformaldehyde solution. After embedding the tissues in paraffin, they were sectioned. The structural integrity of the rat myocardial tissue was assessed using hematoxylin-eosin (HE) staining. TUNEL staining was performed using the ServicebioR DAB(SA-HRP) Tunel Cell Apoptosis Detection Kit (Servicebio, Wuhan, China) to detect apoptotic cells. The stained samples were observed and photographed using a microscope (Nikon Corporation, Kyoto, Japan). The results were quantified as the number of TUNEL-positive cells/total cells × 100%.

### Small-interfering RNA transfection

Small interfering RNA (siRNA) transfection was performed as described in our previous study [38]. Briefly, siRNA targeting IP3R2 (si-IP3R2) and the negative scrambled control (si-NC) were obtained from RuiBo Biology Co., Ltd. (Guangzhou, China). Then, the NRCMs were transfected with si-IP3R2 or si-NC using Lipo3000 Transfection Reagent (Invitrogen) for 24 h.

### Annexin V/propidium iodide (PI) staining

For apoptosis detection, we used an Annexin V-FITC/PI apoptosis detection kit (Sigma) to label the NRCMs with Annexin V and propidium iodide (PI). The cells were seeded at a density of 1 ml/well (approximately 1 × 10^5^ cells) in 12-well plates and treated accordingly as per the protocol. Then, the cells were harvested, washed twice with phosphate-buffered saline (PBS), incubated with the provided dye solution for 10 min at 37 °C and analyzed using a Flow Cytometer (Beckman Coulter, Brea, CA, USA). The different quadrants Q1, Q2, Q3 and Q4 represent necrotic cells, late apoptotic cells, early apoptotic cells and viable cells, respectively. The apoptotic rate of the cells was calculated as the sum of Q2 and Q3.

### Intracellular Ca^2+^ measurement

NRCMs were cultured on confocal dishes and incubated with 3 μM fluo-4/AM dye (Invitrogen, Germany) for 30 min at 37 °C. Subsequently, the NRCMs were rinsed with Ca^2+^-free Tyrode’s solution (composed of 132 mM NaCl, 4.8 mM KCl, 1.2 mM MgCl_2_, 5 mM glucose, 10 mM HEPES, and 1.8 mM CaCl_2_ at pH 7.4) and placed on the stage of a confocal laser scanning microscope (SP5-FCS, Leica). To induce transient intracellular Ca^2+^ release, the NRCMs were stimulated with either 1 μM or 10 μM adenosine 5’-triphosphate (ATP). Alternatively, to induce ER emptying, the NRCMs were stimulated with 2 μM thapsigargin (TG, Sigma-Aldrich). To investigate the role of IP3R in Ca^2+^ release, the NRCMs were pre-treated with 0.1 μM Xestospongin-C (XeC, Sigma-Aldrich) for 15 min before ATP stimulation. To analyze the calcium imaging results, the fluorescence change over time was quantified as F_1_/F_0_, where F_1_ represents the maximal fluorescence, and F_0_ represents basal fluorescence.

### Reverse transcription-quantitative PCR (RT-qPCR)

Total RNA was extracted from myocardial tissues or NRCMs using TRIzol reagent and reversed transcribed into cDNA using PrimeScript RT Master Mix according to the manufacturer’s instructions. RT-qPCR was performed using an SYBR Green PCR Kit (AG, Changsha, China), and the expression of mRNA levels was calculated using the 2^−ΔΔCt^ method. All primers were synthesized by Integrated DNA Technology (Servicebio, Wuhan, China), and the primer information is shown in Table 2.

**Table 2:** Information about the primers we have used in this study.

Gene ID	Gene name	Sequence (5′-3′)
25262	IP3R1	Forward CGAATGGATTTATCAGCACCTT
		Reverse ACCGCATCTGTTGTACTGTTGG
81678	IP3R2	Forward CACTCTGGGAGATAGAGGTGGTT
		Reverse TGAGCATCTCGATAGTCAGGGT
25679	IP3R3	Forward GGAAAGCCAAGCAGACTAAGCA
		Reverse GCCGCTTGTTCACCGTTAAGTAT
287362	NLRP3	Forward CTGAAGCATCTGCTCTGCAACC
		Reverse AACCAATGCGAGATCCTGACAAC
79255	ATF4	Forward AGTCTGCCTTCTCCAGGTGTTC
		Reverse GCTGTCTTGTTTTGCTCCATCTT
29467	CHOP	Forward AAGCCCTCGCTCTCCAGATT
		Reverse CGCTCGTTCTCTTCAGCAAG
24494	IL-1β	Forward CCCTGAACTCAACTGTGAAATAGCA
		Reverse CCCAAGTCAAGGGCTTGGAA
29197	IL-18	Forward ACTGGCTGTGACCCTATCTGTGA
		Reverse TTGTGTCCTGGCACACGTTTC
24383	GAPDH	Forward CTGGAGAAACCTGCCAAGTATG
		Reverse GGTGGAAGAATGGGAGTTGCT

### Western blot

Whole-cell and tissue proteins were extracted using RIPA strong lysis buffer (Beyotime, China). Protein samples were standardized to 20 μg using 4× SDS loading buffer (Takara, Japan) and denatured at 100 °C for 10 min. After performing SDS-PAGE electrophoresis, the proteins were transferred onto PVDF membranes (Millipore) and blocked with 5% skimmed milk buffer at room temperature for 1 h. The membranes were then incubated with primary antibodies overnight at 4 °C, followed by incubation with secondary antibodies at room temperature for 1 h. The primary antibodies used were as follows: IP3R2 (sc-398434, Santa), NLRP3 (15101, CST), eIF2α (9722, CST), p-eIF2α (9721, CST), ATF4 (11815, CST), CHOP (2895, CST), GSDMD-FL (39754, CST), GSDMD-NT (AF4012, Affinity), IL-1β (ab283818, Abcam), IL-18 (67775, CST), Caspase-1 p10 (BS5641, Bioworld), and GAPDH (Proteintech, 60004). The secondary antibodies used were anti-rabbit or anti-mouse antibodies (160491 or 159908, Jackson). Finally, the protein bands were visualized using an ECL kit (BL520A, Biosharp) and quantified using ImageJ software.

### Immunofluorescent staining of ASC specks

The NRCMs were seeded on confocal dishes and pre-treated with MCC950 (10 μM) for 1 h before being stimulated with LPS (2 μg/ml) for 24 h. After that, NRCMs were fixed in 4% paraformaldehyde and permeabilized with 0.1% Triton X-100. NRCMs were immunofluorescently stained overnight at 4 °C with anti-ASC antibody (Proteintech, 67494) after blocking with 5% BSA. After incubation with goat anti-mouse 488 secondary antibody (Invitrogen, A-10680) for 1 h at room temperature, NRCMs were stained with DAPI (Sigma, F6057) for nucleus staining. Fluorescent images were acquired using Zeiss LSM900 confocal microscope (Germany).

### Statistical analysis

The data are presented as means ± SEM. Statistical analyses were performed using the SPSS v22.0 software, and the results were visualized using GraphPad Prism 8.0. Differences between two groups were analyzed using unpaired Student’s *t* test, while comparisons among multiple groups were conducted using one-way ANOVA. *P* values < 0.05 were considered statistically significant.

### Supplementary information

Supplementary Figure S1

Supplementary Figure S2

Supplementary Figure S3

Supplementary Figure Legends

Original full length western blots

## Data Availability

The data generated during this study have been included in the published article as well as its Supplementary Data file. If necessary, all data can be obtained from the corresponding author.
