# Supplementary figures and images for: IP3R2-mediated Ca2+ release promotes LPS-induced cardiomyocyte pyroptosis via the activation of NLRP3/Caspase-1/GSDMD pathway
Source: Cell Death Discov. 2024 Feb 20;10:91. doi: 10.1038/s41420-024-01840-8 (PMC10879485; doi:10.1038/s41420-024-01840-8)

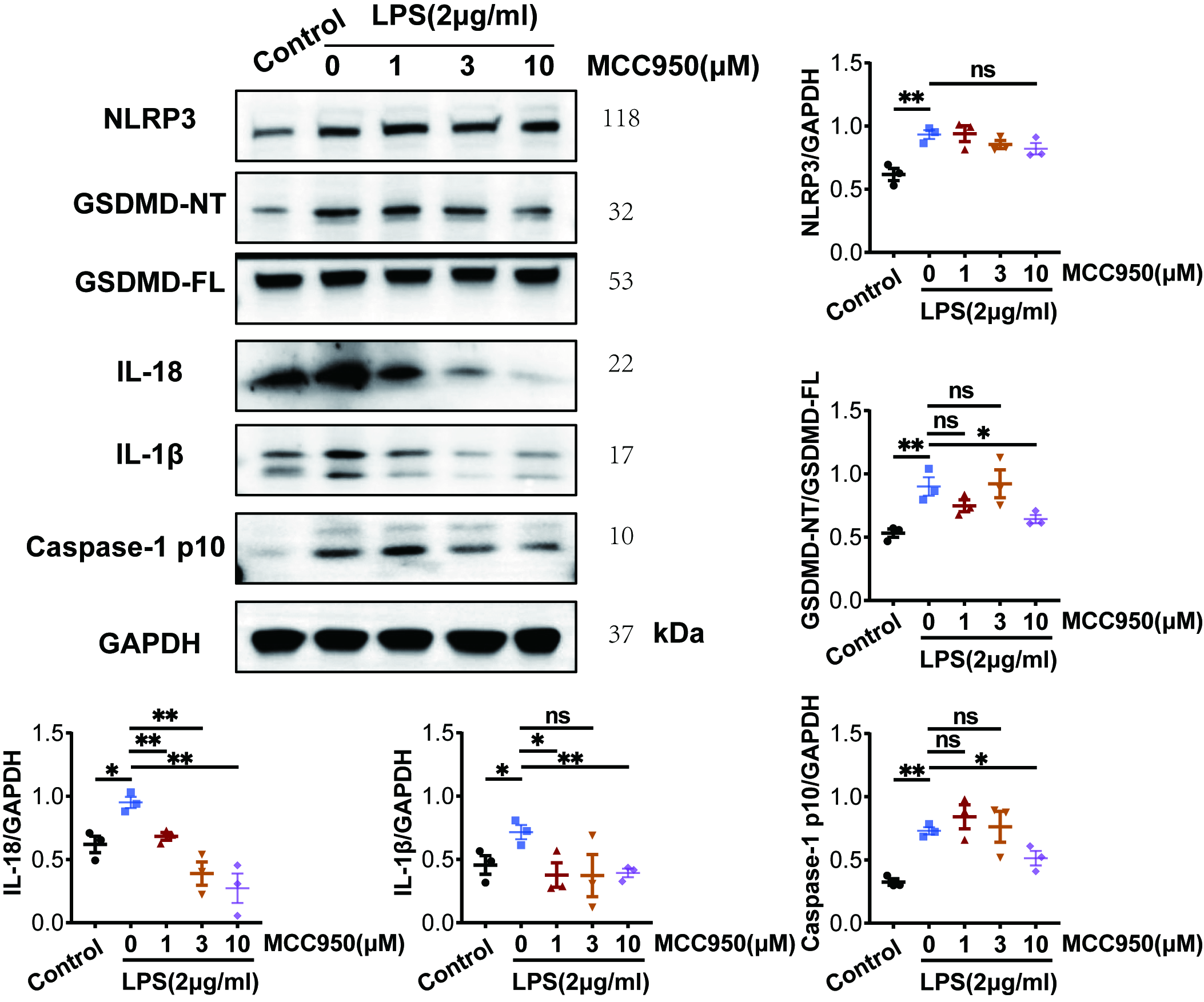

Supplement: Supplementary file 1 — Supplementary Figure S1 [file 41420_2024_1840_MOESM1_ESM.tif]

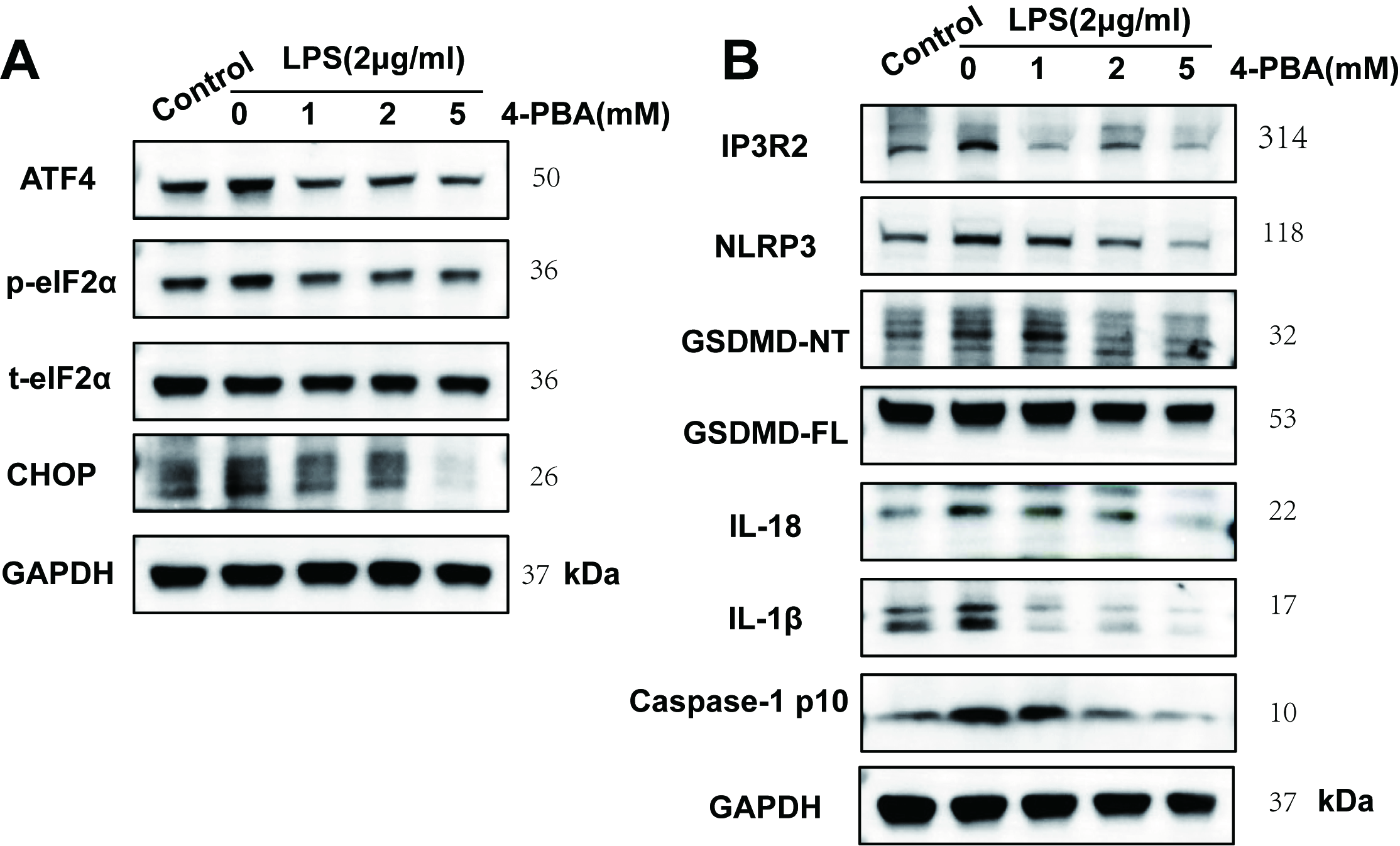

Supplement: Supplementary file 2 — Supplementary Figure S2 [file 41420_2024_1840_MOESM2_ESM.tif]

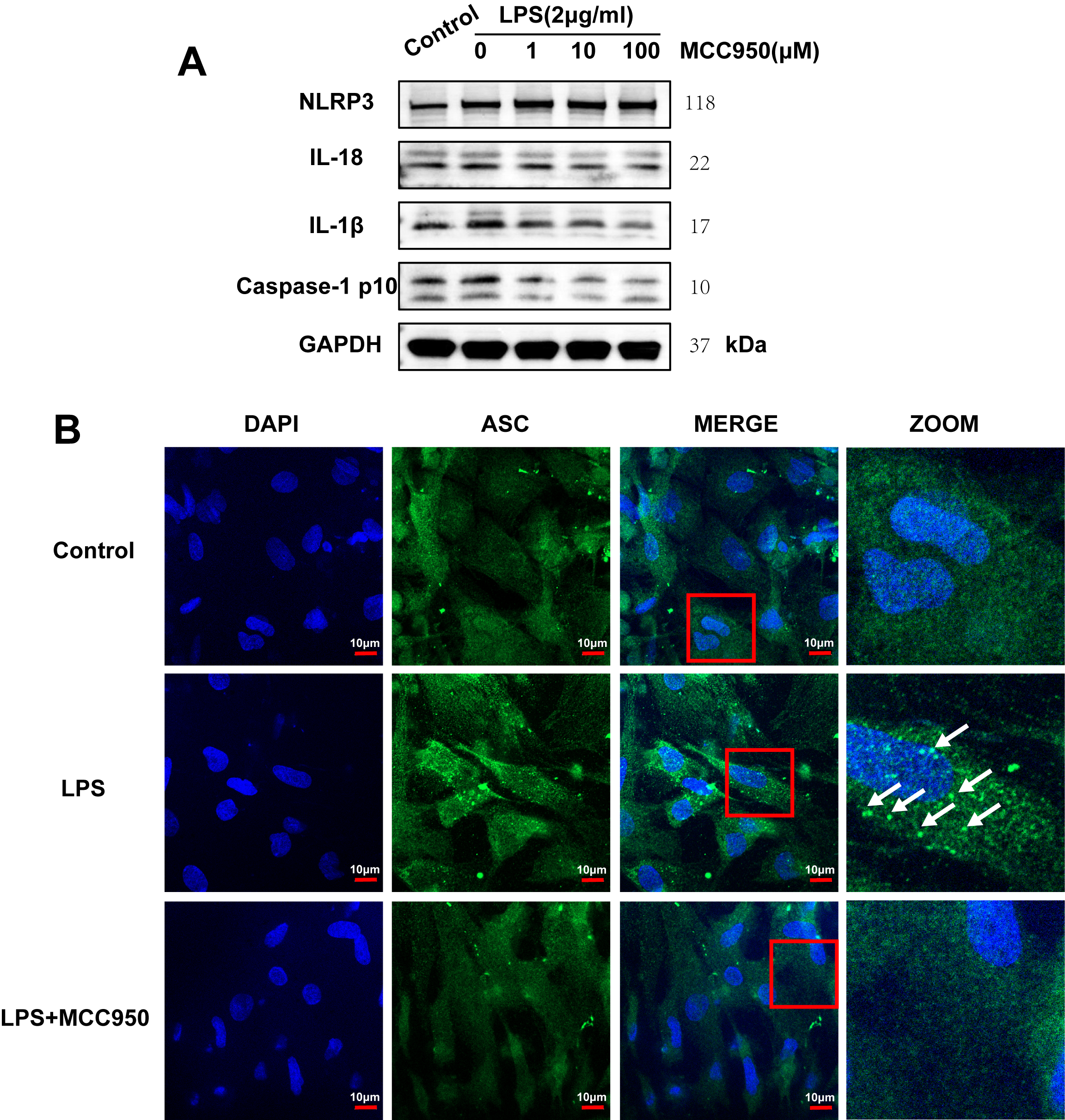

Supplement: Supplementary file 3 — Supplementary Figure S3 [file 41420_2024_1840_MOESM3_ESM.tif]
