# Supplementary material for: IP3R2-mediated Ca2+ release promotes LPS-induced cardiomyocyte pyroptosis via the activation of NLRP3/Caspase-1/GSDMD pathway
Source: Cell Death Discov. 2024 Feb 20;10:91. doi: 10.1038/s41420-024-01840-8 (PMC10879485; doi:10.1038/s41420-024-01840-8)
Supplement: Supplementary file 4 — Supplementary Figure Legends [file 41420_2024_1840_MOESM4_ESM.docx]

**Supplementary Figure Legends**

**Supplementary Figure S1. MCC950 dose dependently inhibited the expression of GSDMD-NT, mature Caspase-1, IL-1β, and IL-18, but not NLRP3 in NRCMs induced by LPS.** Western blotting images and the summarized data showing the effect of MCC950 (1, 3, 10μM) on the protein expression of NLRP3, GSDMD, Caspase-1 p10, IL-18 and IL-1β in NRCMs-induced by LPS (n = 3). Data are shown as Mean ± SEM. **P* < 0.05, ***P* < 0.01. ns: no significant statistical difference.

**Supplementary Figure S2.** **Pre-experiment to determine 4-PBA intervention concentration in NRCMs-induced by LPS.** (A) Western blotting images showing the effect of 4-PBA (1, 2, 5 mM) on the ER stress markers eIF2α and CHOP expression in the NRCMs induced with LPS. The induction of p-eIF2α and CHOP by ER stress was suppressed by 4-PBA in a dose-dependent manner. (B) Western blotting images showing the effect of 4-PBA (1, 2, 5mM) on the protein expression of NLRP3, GSDMD-NT, Caspase-1 p10, IL-18 and IL-1β in NRCMs-induced by LPS. The data showed that 4-PBA inhibited NLRP3 inflammasome activation in LPS induced NRCM at 5 mM concentrations.

**Supplementary Figure S3. MCC950 could inhibit NLRP3 inflammasome activation through blocking ASC oligomerization induced by LPS in NRCMs.** (A) Western blotting images showing the MCC950 (1, 10, 100μM) dose dependently inhibited the expression of mature Caspase-1, IL-1β, and IL-18, but not NLRP3 in NRCMs induced by LPS. (B) Immunofluorescent staining shows MCC950 (10μM) blocking ASC specks formation in NRCMs induced by LPS. The arrows indicate ASC specks.
