# Supplementary material for: IP3R2-mediated Ca2+ release promotes LPS-induced cardiomyocyte pyroptosis via the activation of NLRP3/Caspase-1/GSDMD pathway
Source: Cell Death Discov. 2024 Feb 20;10:91. doi: 10.1038/s41420-024-01840-8 (PMC10879485; doi:10.1038/s41420-024-01840-8)
Supplement: Supplementary file 5 — Original full length western blots [file 41420_2024_1840_MOESM5_ESM.pdf]

Figure1 E

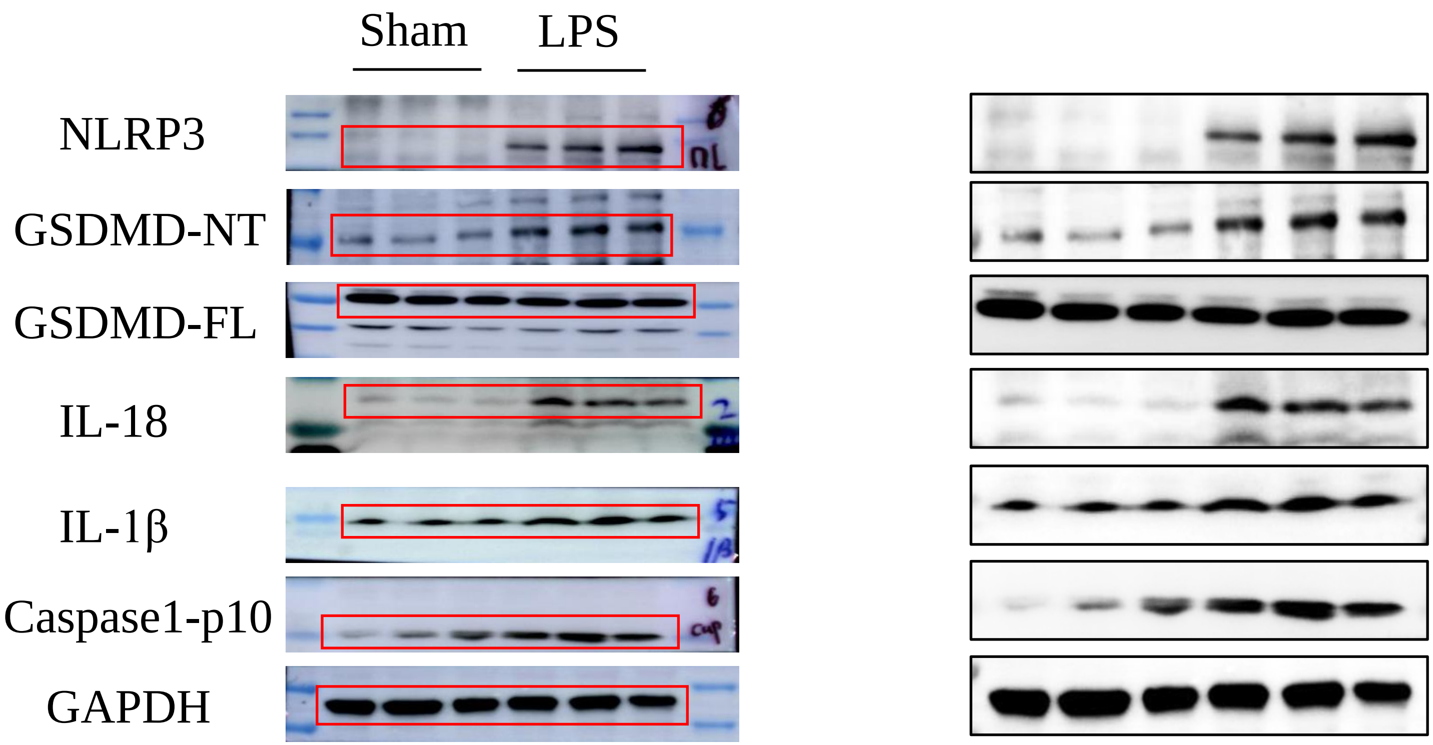

Sham
LPS
NLRP3
GSDMD-NT
GSDMD-FL
IL-18
IL-1β
Caspase1-p10
GAPDH

Figure2 B

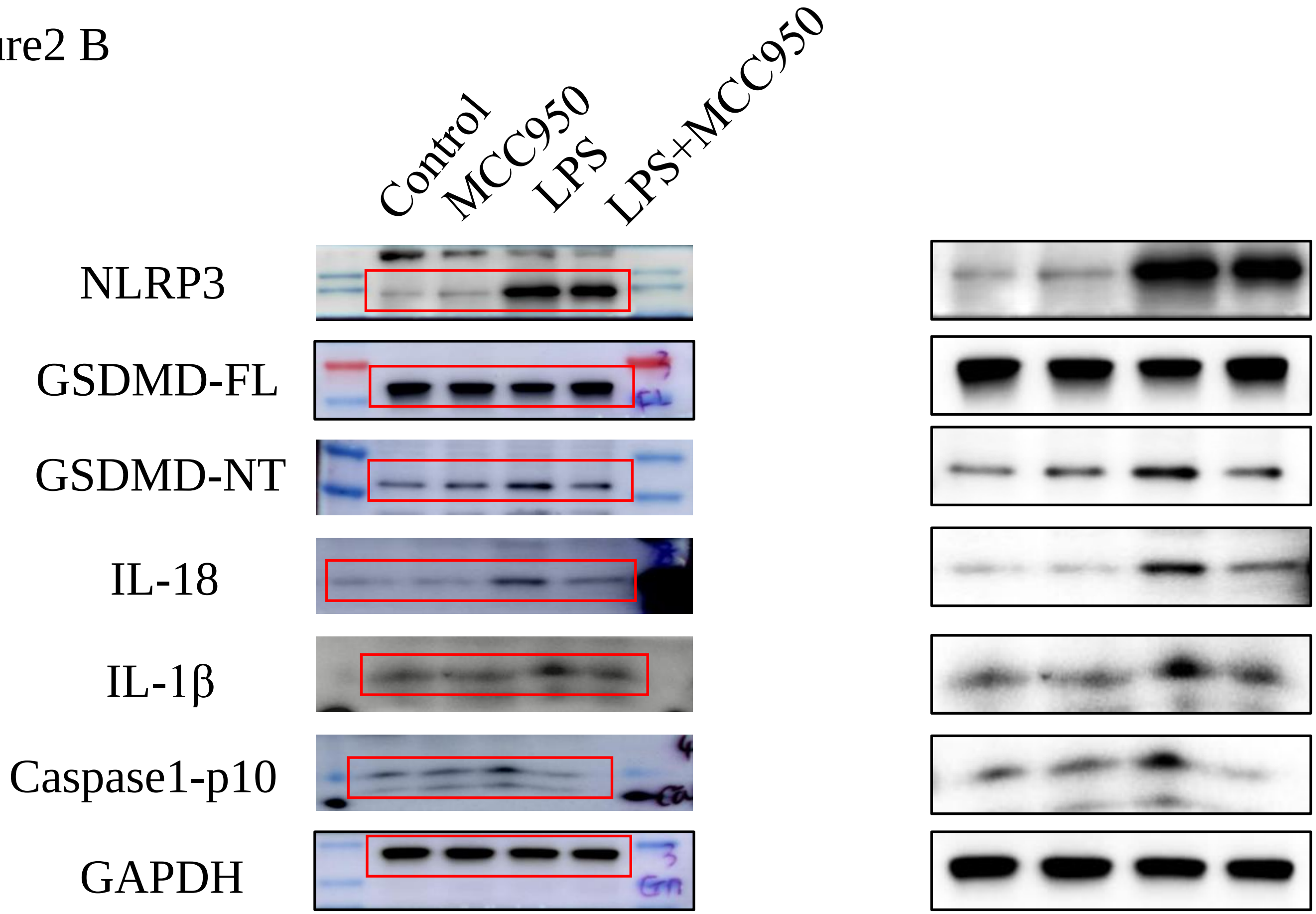
Control
MCC950
LPS
LPS+MCC950
NLRP3
GSDMD-FL
GSDMD-NT
IL-18
IL-1β
Caspase1-p10
GAPDH

Figure3 D

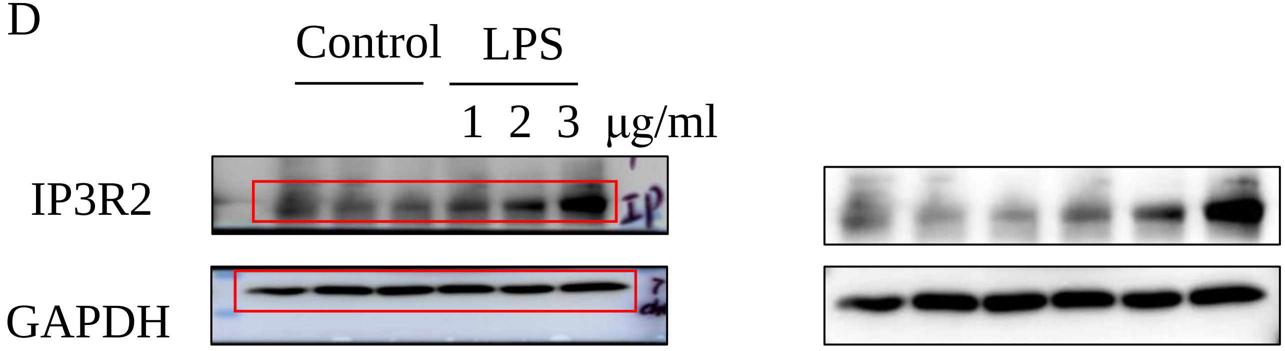

Figure3 E

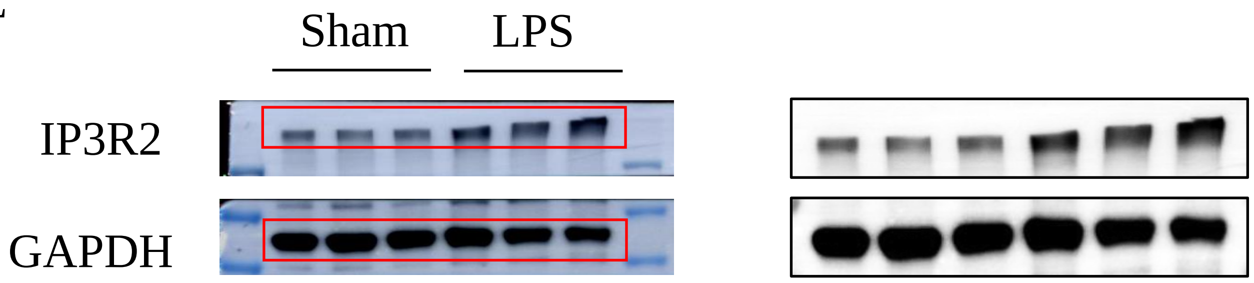

Figure4 C

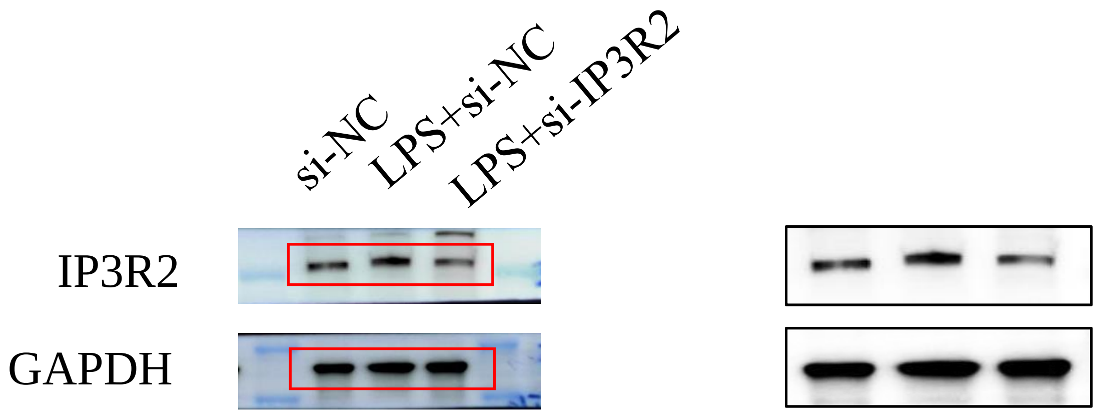

Figure4 I

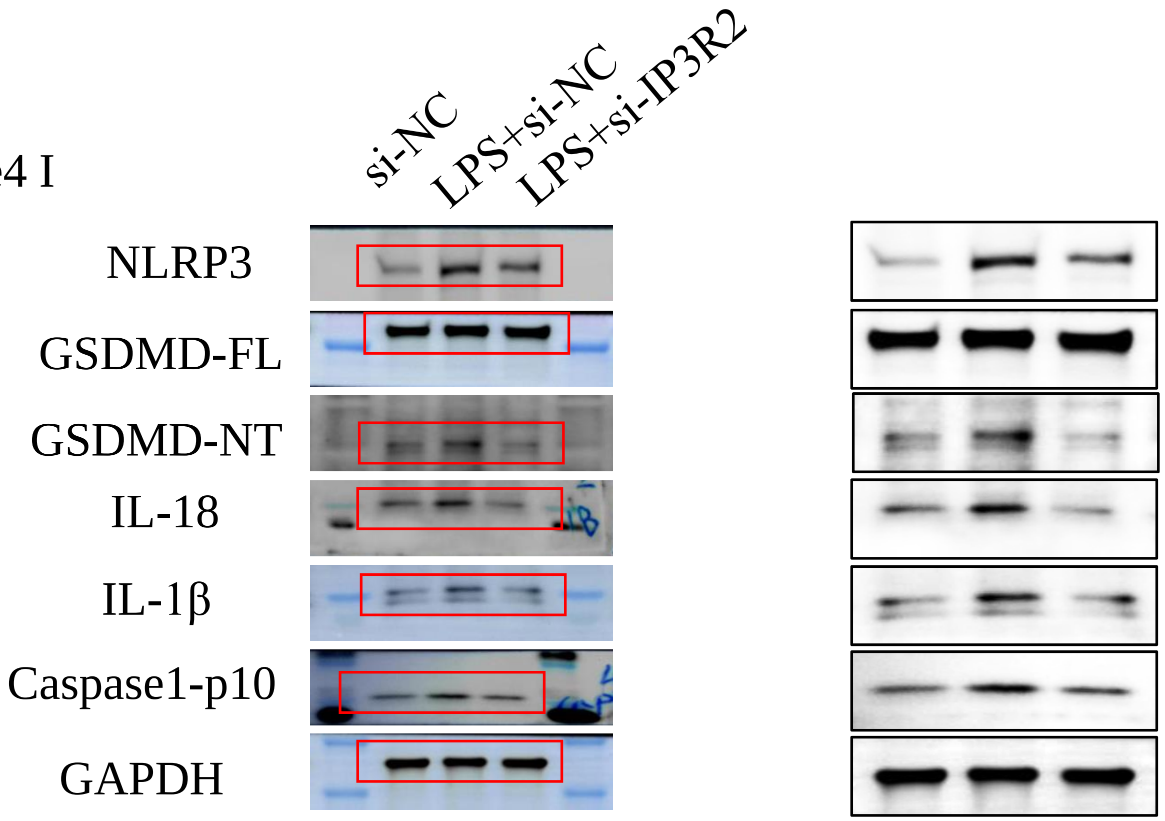

Figure5 C

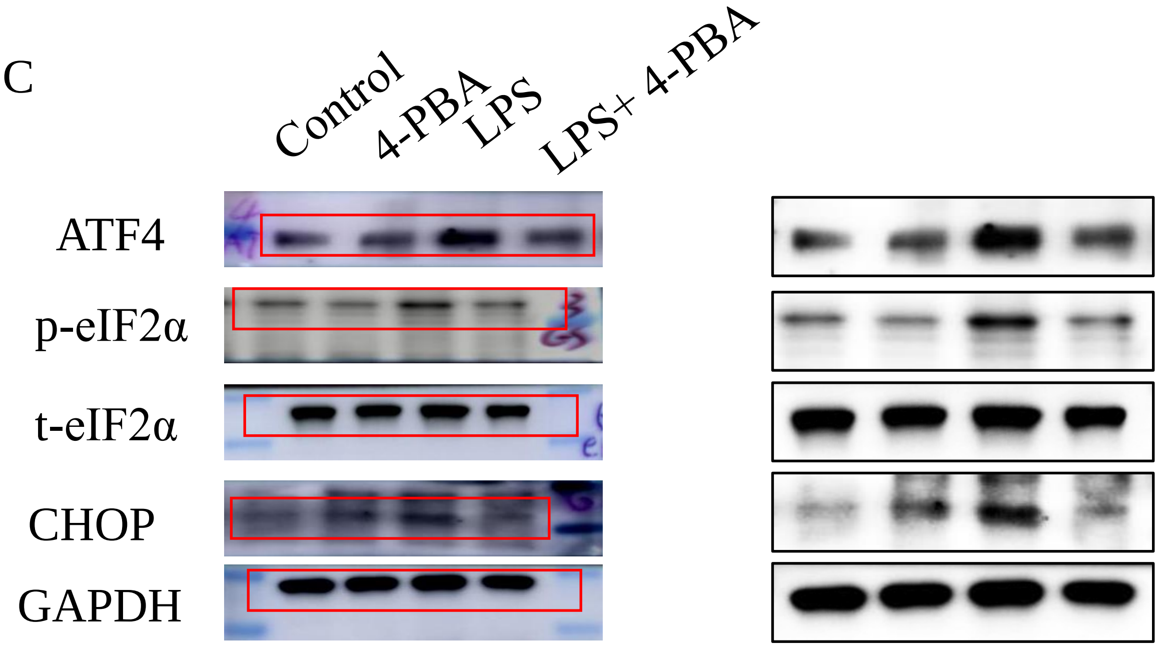

Figure5 E

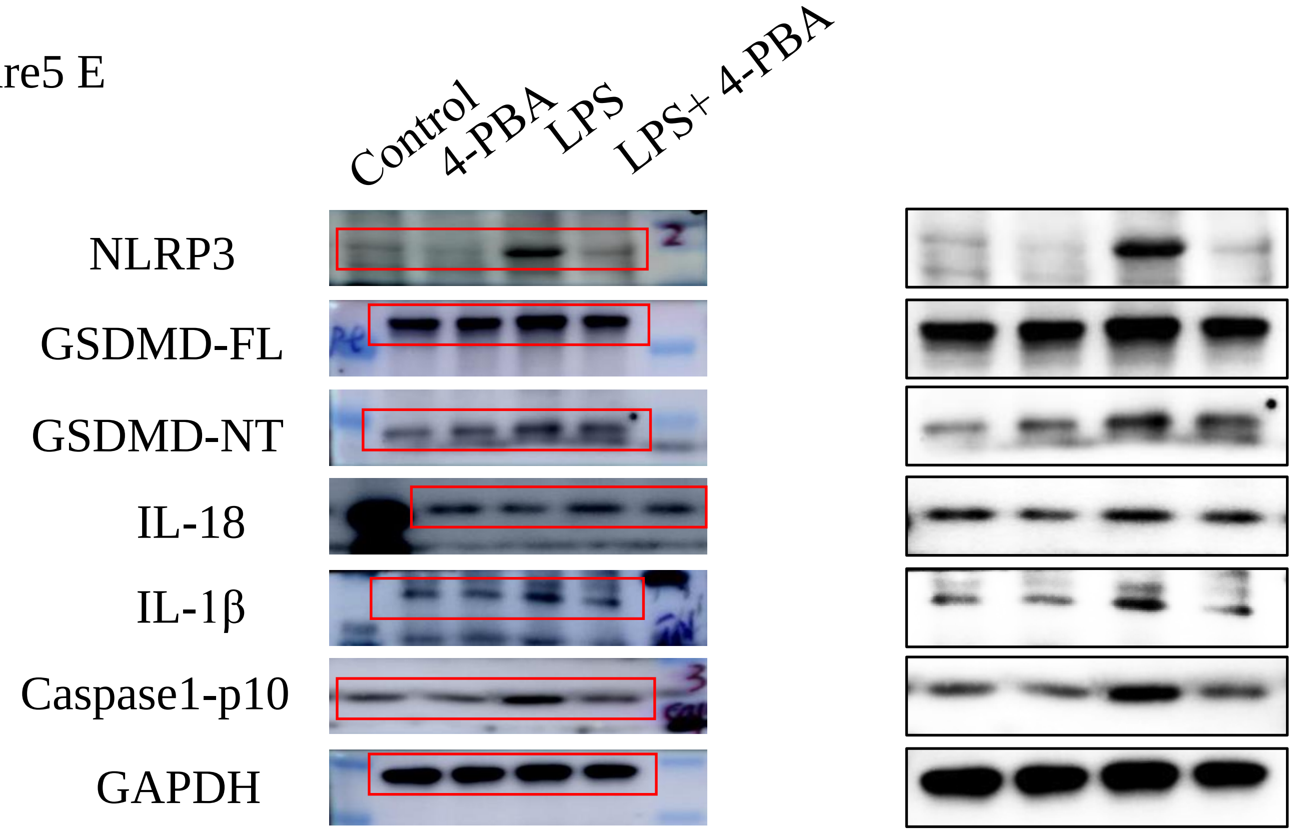
Control
4-PBA
LPS
LPS+ 4-PBA
NLRP3
GSDMD-FL
GSDMD-NT
IL-18
IL-1β
Caspase1-p10
GAPDH

Figure6 A

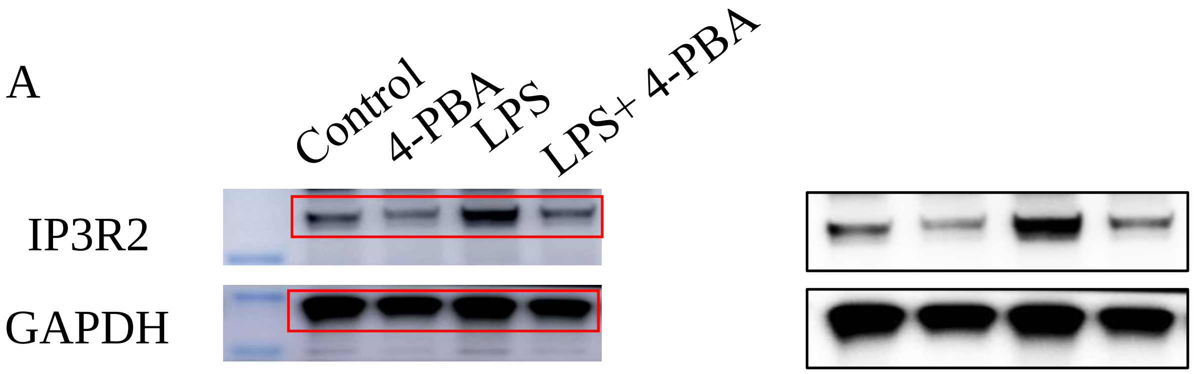

Figure6 E

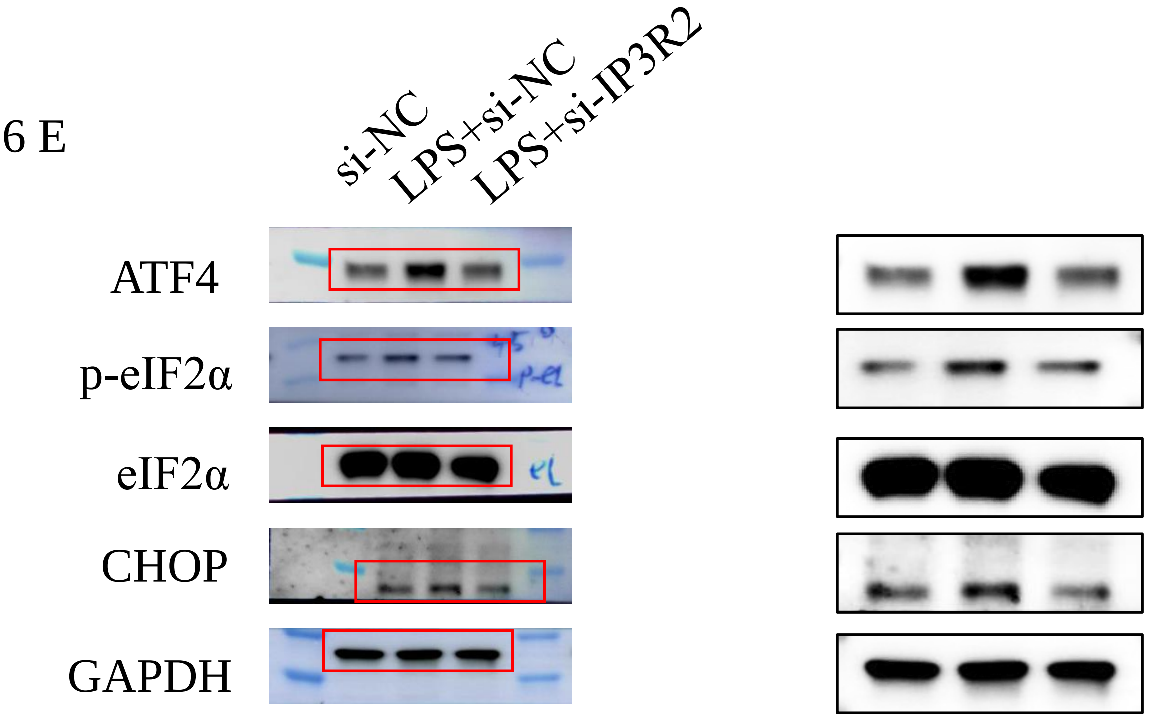

Figure S1

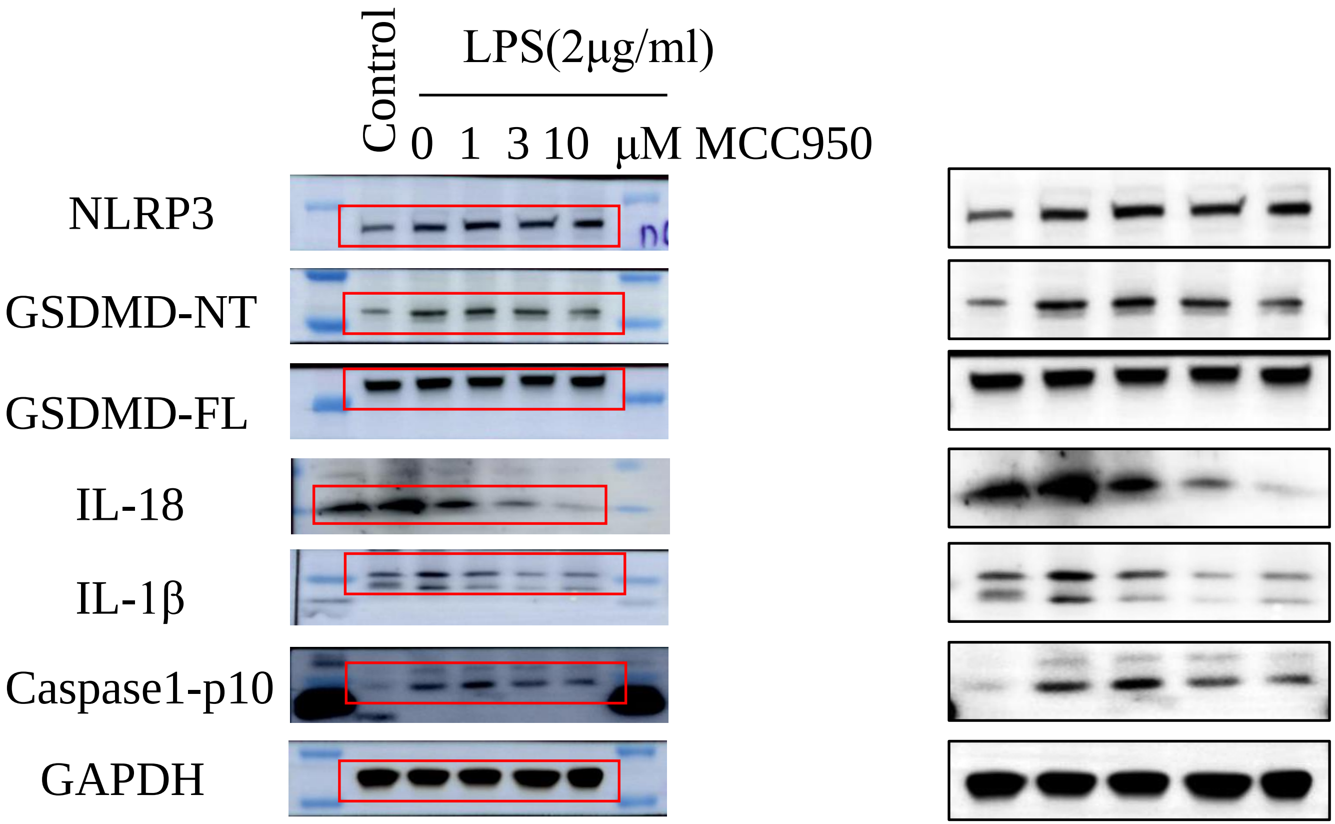

Control
LPS(2μg/ml)
0 1 3 10 μM MCC950
NLRP3
GSDMD-NT
GSDMD-FL
IL-18
IL-1β
Caspase1-p10
GAPDH

Figure S2 A

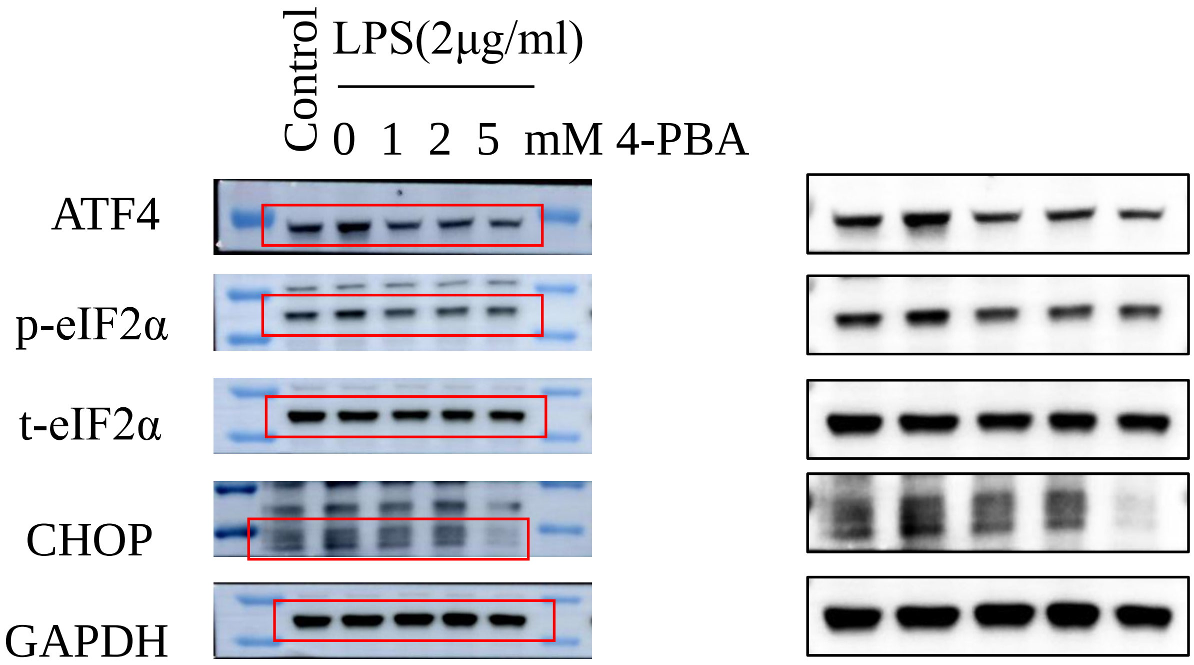
Control
LPS(2μg/ml)
0 1 2 5 mM 4-PBA
ATF4
p-eIF2α
t-eIF2α
CHOP
GAPDH

Figure S2 B

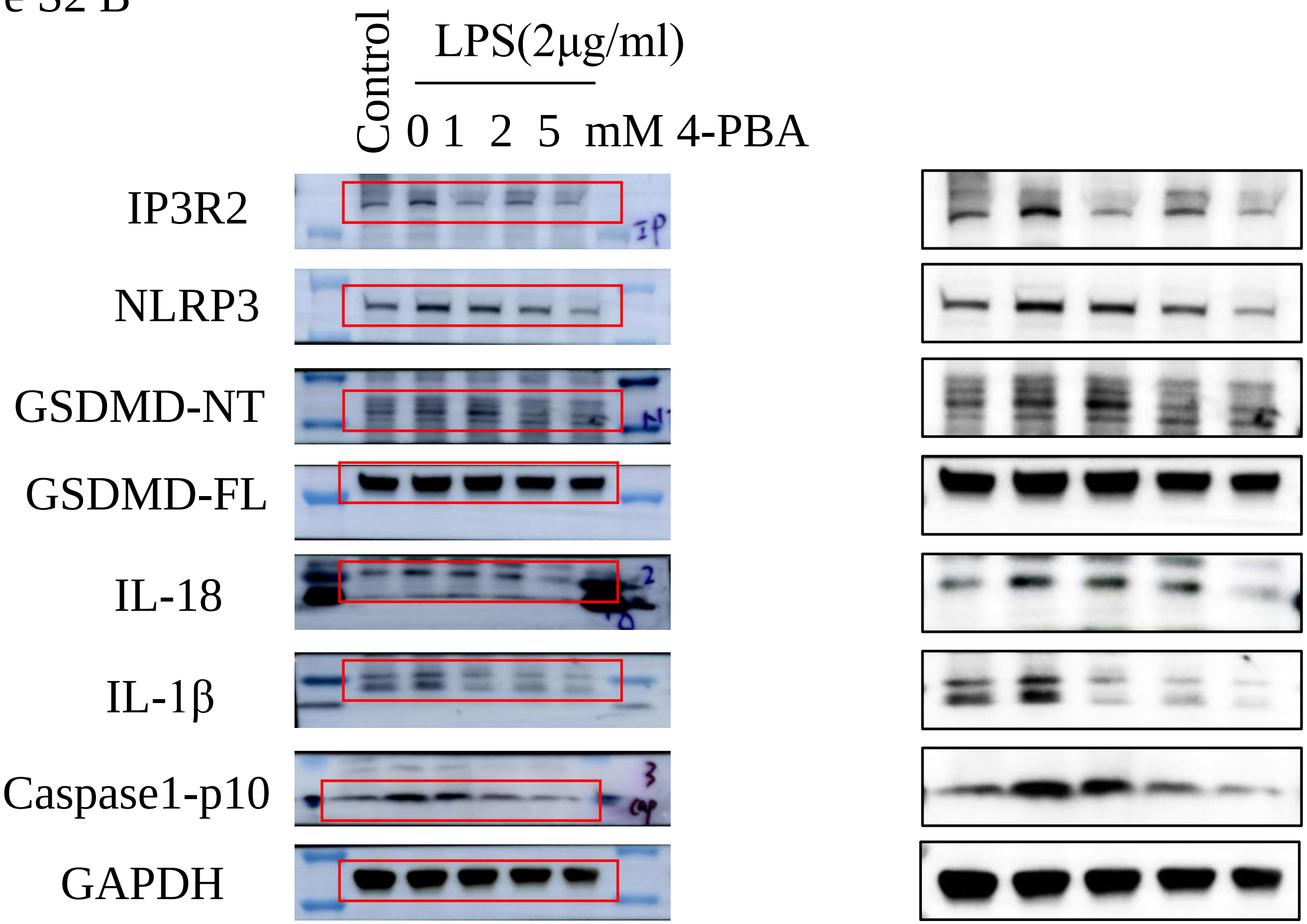

Control
LPS(2μg/ml)
0 1 2 5 mM 4-PBA
IP3R2
NLRP3
GSDMD-NT
GSDMD-FL
IL-18
IL-1β
Caspase1-p10
GAPDH

Figure S3

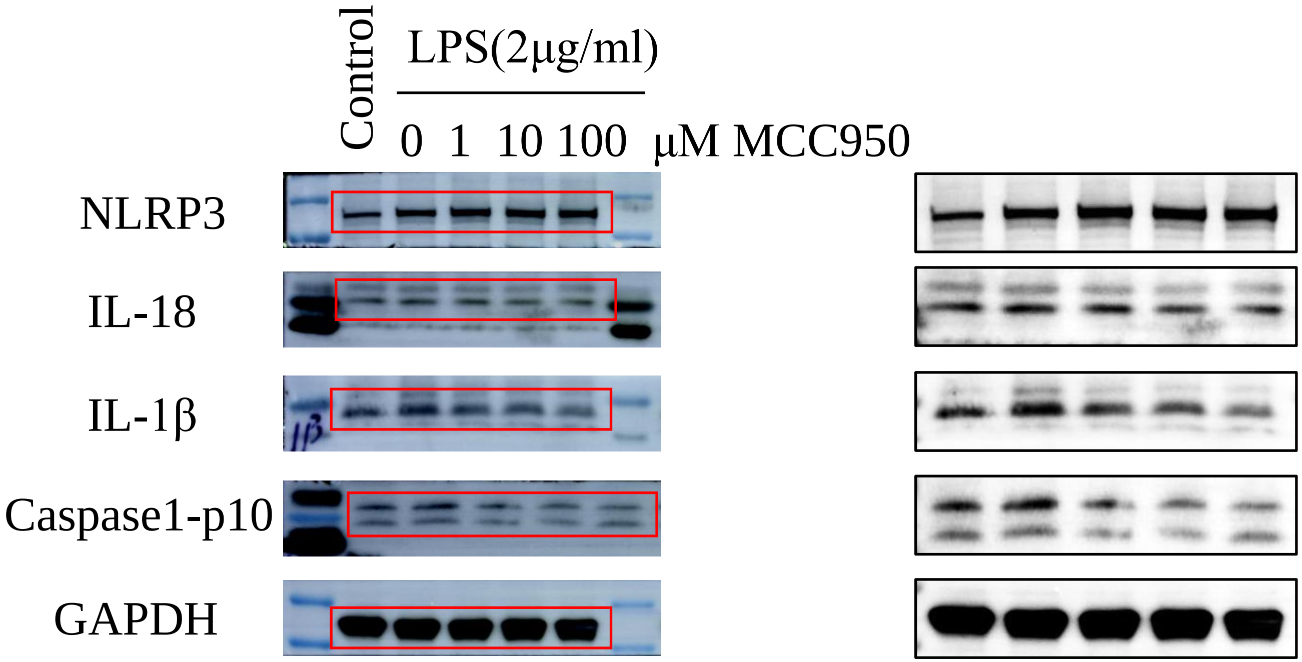

Control
LPS(2μg/ml)
0 1 10 100 μM MCC950
NLRP3
IL-18
IL-1β
Caspase1-p10
GAPDH
